# Differences Between Autistic and Non-Autistic Adults in the Recognition of Anger from Facial Motion Remain after Controlling for Alexithymia

**DOI:** 10.1007/s10803-021-05083-9

**Published:** 2021-05-28

**Authors:** Connor T. Keating, Dagmar S. Fraser, Sophie Sowden, Jennifer L. Cook

**Affiliations:** School of Psychology, University of Birmingham, Birmingham, UK

**Keywords:** Autism spectrum disorder, Facial expression, Emotion recognition, Movement kinematics, Alexithymia

## Abstract

To date, studies have not established whether autistic and non-autistic individuals differ in emotion recognition from facial motion cues when matched in terms of alexithymia. Here, autistic and non-autistic adults (N = 60) matched on age, gender, non-verbal reasoning ability and alexithymia, completed an emotion recognition task, which employed dynamic point light displays of emotional facial expressions manipulated in terms of speed and spatial exaggeration. Autistic participants exhibited significantly lower accuracy for angry, but not happy or sad, facial motion with unmanipulated speed and spatial exaggeration. Autistic, and not alexithymic, traits were predictive of accuracy for angry facial motion with unmanipulated speed and spatial exaggeration. Alexithymic traits, in contrast, were predictive of the magnitude of *both* correct and incorrect emotion ratings.

**Supplementary Information:**

The online version contains supplementary material available at 10.1007/s10803-021-05083-9.

## Introduction

Autism spectrum disorder (ASD) is a neurodevelopmental disorder, characterized by difficulties in social communication, and restricted and repetitive interests (American Psychiatric Association, 2013). Since the ability to infer emotion from facial expressions is important for social interaction, emotion recognition has long been suspected as a difficulty in ASD (Hobson, 1986). However, whilst many studies suggest a disparity in the facial emotion recognition ability of autistic^1^ and non-autistic individuals (Ashwin et al., 2006; Dziobek et al., 2010; Lindner & Rosén, 2006; Philip et al., 2010), there have been inconsistent findings, ranging from no differences between these individuals to large disparities (see Harms et al., 2010, Keating & Cook, 2020, and Uljarevic & Hamilton, 2013 for reviews). Consequently, the question of whether autistic individuals exhibit atypical facial emotion recognition has been debated for over 30 years.

The most recent contributions to this debate claim that it is not autism per se that is linked to emotion recognition atypicalities but rather alexithymia (Bird & Cook, 2013; Kinnaird et al., 2019; Oakley et al., 2016; Poquérusse et al., 2018). Alexithymia is a subclinical condition, characterized by difficulties identifying and expressing emotions (Nemiah et al., 1976), which is often comorbid with ASD (in the neurotypical population the prevalence of alexithymia is 4.89%, and in autistic populations the prevalence of alexithymia is 49.93% (Kinnaird et al., 2019)). Cook et al. (2013) demonstrated that continuous measures of alexithymic, but not autistic, traits are predictive of poorer facial emotion recognition from static face images. Furthermore, when groups are matched in terms of alexithymia, autistic and non-autistic adults perform comparably with respect to the recognition of emotion (Cook et al., 2013). Similarly, Milosavljevic et al., (2016) demonstrated lower emotion recognition scores—again from static face images—for autistic adolescents high in alexithymia relative to those low in alexithymia. Consequently, Bird and Cook (2013) propose ‘the alexithymia hypothesis’: autistic individuals’ difficulties in emotion-processing, including facial emotion recognition, are caused by co-occurring alexithymia not ASD.

To date, the majority of studies that have reported that atypical facial emotion processing is related to alexithymia, not autism, have focused on the recognition of emotion from *static* face images, and have thus overlooked the inherently dynamic nature of facial expressions (Kilts et al., 2003; Sato et al., 2004). Dynamic faces carry both spatial information about the configuration of facial features relative to each other and information about the kinematics (e.g., speed) of movement of facial features (Dobs et al., 2018). Recent developments in the face processing literature emphasize the importance of both kinematic and spatial cues in *non-autistic* facial emotion recognition. Most notably, Sowden et al. (2021) manipulated point-light face (PLF) stimuli (a series of white dots on a black background that convey biological motion and eliminate contrast, texture, colour and luminance cues) such that expressions of happiness, anger and sadness were reproduced at 50%, 100% and 150% of their normal speed, and at 50%, 100% and 150% of their normal range of spatial movement (e.g., at the 150% spatial level a smile would be 50% bigger / more exaggerated than normal). Sowden et al. (2021) found that the emotion recognition accuracy of non-autistic participants was modulated as a function of both spatial and kinematic manipulation. Specifically, when expressions were reduced in their speed and spatial extent (i.e., at the 50% level), participants were less accurate in their labelling of angry and happy expressions and more accurate for sad expressions. Conversely, when expressions were played with exaggerated spatial movement and greater speed (i.e., at the 150% level), participants displayed higher accuracy for angry and happy expressions and lower accuracy for sad expressions (Sowden et al., 2021). Thus, accuracy for labelling high arousal emotions (happy and angry) is improved when the stimulus is faster and more spatially exaggerated, whereas labelling of low arousal emotions (sad) is impaired. Recent literature therefore highlights that, for non-autistic individuals, both spatial and kinematic facial cues contribute to emotion recognition accuracy.

Although dynamic information is particularly important in real life processing of facial expressions (Krumhuber et al., 2013), to the best of our knowledge, there are no studies that have investigated autistic versus non-autistic recognition of emotion *from dynamic facial motion stimuli* (e.g., PLFs) whilst controlling for the influence of alexithymia. There are, however, some studies that have compared autistic and non-autistic processing of full (i.e., not degraded) dynamic facial expressions without controlling for alexithymia. For example, Sato et al. (2013) demonstrated that for non-autistic adults reducing the speed of movement of facial morph stimuli^2^ reduced naturalness ratings, however, for autistic adults the effect of speed on naturalness ratings was significantly weaker. Sato and colleagues’ results thus demonstrate differences, between autistic and non-autistic adults, in the effects of manipulating facial kinematics. However, it remains to be seen whether these differences would persist if the groups were matched in terms of alexithymia. To the best of our knowledge, only one study has examined the contribution of autistic *and* alexithymic traits to dynamic emotion recognition (Ola & Gullon-Scott, 2020). The findings of this study support the alexithymia hypothesis: high alexithymic, but not autistic, traits were associated with less accurate facial expression recognition (Ola & Gullon-Scott, 2020). However, this study has two important limitations. First, only female participants were recruited. Since autistic males comprise three quarters of the ASD population (Loomes et al., 2017), and likely differ in behavioural phenotype (Ketelaars et al., 2016; Rivet & Matson, 2011), one must be cautious about extrapolating the findings to autistic males. Second, the authors did not recruit a non-autistic control group. Consequently, they were not able to explore whether autistic versus non-autistic group differences in dynamic emotion recognition remain after controlling for alexithymia. That is, although the authors were able to show that *some* difficulties with emotion recognition from dynamic stimuli were associated with alexithymia, one cannot conclude from this study that there are *no* differences with respect to emotion recognition from dynamic stimuli that are specifically associated with ASD.

The primary aim of the current study was to investigate whether autistic and non-autistic adults would exhibit differences in the recognition of emotion from facial motion cues when the groups were matched in terms of alexithymia. To address this aim we employed the paradigm developed by Sowden et al. (2021) which uses PLF stimuli to represent emotional expressions in terms of the movement of facial landmarks. More specifically, male and female autistic adults and non-autistic controls rated the emotion expressed by PLF stimuli that had been manipulated such that expressions of happiness, anger and sadness were reproduced at 50%, 100% and 150% of their normal speed and spatial extent. The groups were matched in terms of their scores on a self-report measure of alexithymia. We predicted that emotion recognition accuracy would be affected by both kinematic and spatial manipulation and that these effects would not interact with group, but rather that Bayesian statistics would provide support for the null hypothesis that the alexithymia-matched groups perform comparably. Given that we had considerable variation in alexithymic traits, a secondary aim of our study was to explore whether the effects of the spatial and kinematic manipulation on emotion recognition accuracy covaried with scores on the self-report alexithymia measure.

## Method

### Participants

The chosen sample size is based on an a priori power analysis conducted using GLIMMPSE (Kreidler et al., 2013), which focused on replicating the primary results from Sowden et al., (2021) in the control group (the emotion × spatial and emotion × kinematic interactions). Using data from Sowden et al., (2021), 8 participants are required in the control group in order to have 95% power to detect an effect size of 0.70 (*η*_*P*_^*2*^) at alpha level 0.01 for the emotion × spatial interaction. Moreover, 11 participants are required in the control group in order to have 95% power to detect an effect size of 0.53 (*η*_*P*_^*2*^) for the emotion × kinematic interaction at alpha level 0.01. However, Button et al. (2013) argue that effect size estimates are commonly inflated (“the winners curse”), and that there is “a common misconception that a replication study will have sufficient power to replicate an initial finding if the sample size is similar to that in the original study”. Accordingly, we planned to recruit a larger number of participants (N = 30 per group; almost triple the largest sample size generated in our power calculations), in order to obtain adequate power. We pre-registered this sample size via the Open Science Framework (https://osf.io/kpefz).

Sixty individuals, 31 with an ASD diagnosis and 29 non-autistic controls, participated in the study (see Supplementary Information A for ethnicity information). Participants were matched for age, gender, non-verbal reasoning (NVR), as measured by the Matrix Reasoning Item Bank (MaRs-IB; Chierchia et al., 2019), and alexithymia, as measured by the 20-item Toronto Alexithymia Scale (TAS-20; Bagby et al., 1994). The ASD group had significantly higher Autism Quotient (AQ; Baron-Cohen et al., 2001) scores (see Table 1). The level of autistic characteristics of those in the ASD group was assessed using the Autism Diagnostic Observation Schedule (version 2, ADOS-2; Lord et al., 2012). The mean total ADOS-2 score in the ASD group was 10.59 (see Supplementary Information B for information on the quantity of participants that met criteria for diagnosis). The MaRs-IB was used to match participants on the basis that the PLF task relies on non-verbal reasoning ability and, with respect to participant matching, task specific measures of intelligence/ability have been argued to be more appropriate than general measures (Mottron, 2004). A total of four participants (three in the ASD group and one in the control group) had AQ or TAS-20 scores over two standard deviations from their group mean. Since the general pattern of results was unaffected by their removal, these participants were included in the final analysis.

**Table 1:** Means, standard deviations and group differences of participant characteristics

	Control group (n = 29)	ASD group (n = 31)	Significance
Gender	11 Female, 17 male, 1 other	14 Female, 16 male, 1 other	p = 0.850
Age	28.81 (9.54)	30.14 (9.08)	p = 0.581
NVR	62.91 (15.17)	57.05 (17.90)	p = 0.178
TAS-20	55.66 (13.57)	59.74 (13.14)	p = 0.241
AQ	19.86 (7.44)	32.52 (10.21)	p < 0.001
ADOS-2	N/A	10.32 (4.76)	N/A

In the central columns, means are followed by standard deviations in parentheses

Twenty-two of the 31 ASD participants were recruited via an existing autism research database kept by the Birmingham Psychology Autism Research Team (B-PART). The control and remaining nine ASD participants were recruited via social media (Facebook and Twitter) and Prolific—an online recruitment platform. All participants in the ASD group had previously received a clinical diagnosis of ASD from a qualified clinician.

### Materials and Stimuli

#### PLF Stimuli

The PLF task was an adapted version of that developed by Sowden and colleagues (2021) which was re-programmed in Gorilla.sc (Anwyl-Irvine et al., 2020) to facilitate online testing. The same instructions, stimulus videos, and rating scales were used as in the original study. The stimulus videos comprised dynamic PLF stimuli, created from videos of four actors (two male, two female) verbalising sentences (“My name is John and I’m a scientist”) whilst posing three target emotions (angry, happy and sad). PLFs were adapted (see Sowden et al., for further detail) to achieve three spatial movement levels, ranging from decreased to increased spatial movement (S1: 50% spatial movement; S2: 100% spatial movement; S3: 150% spatial movement), and three kinematic levels, ranging from reduced to increased speed (K1: 50% original stimulus speed; K2: 100% original stimulus speed; K3—150% of the original stimulus speed). Consequently, there were 9 manipulations per emotion (e.g., (1) S1, K1, (2) S2, K1, (3) S3, K1, (4) S1, K2, (5) S2, K2, (6) S3, K2, (7) S1, K3, (8), S2, K3, (9) S3, K3).

### Autistic Traits

The autistic traits of all ASD and control participants were assessed via the 50-item Autism Quotient (Baron-Cohen et al., 2001). This self-report questionnaire is scored on a range from 0 to 50, with higher scores representing higher levels of autistic characteristics. The AQ assesses five different domains relevant for ASD traits (attention switching, attention to detail, communication, social skill and imagination). The AQ has been widely used in both the general and the autistic population (Ruzich et al., 2015, 2016), and has strong psychometric properties, including internal consistency (α ≥ 0.7) and test–retest reliability (r ≥ 0.8; Stevenson & Hart, 2017).

### Alexithymia

Alexithymia was measured via the 20-item Toronto Alexithymia Scale (Bagby et al., 1994). The TAS-20 comprises 20 items rated on a five-point Likert scale (ranging from 1, strongly disagree, to 5, strongly agree). Total scores on the TAS-20 can range from 20 to 100, with higher scores indicating higher levels of alexithymia. The TAS-20 is the most popular self-report tool for alexithymia and boasts good internal consistency (α ≥ 0.7) and test–retest reliability (r ≥ 0.7) (Bagby et al., 1994; Taylor et al., 2003).

### Non-verbal reasoning

Non-verbal reasoning was assessed via the Matrix Reasoning Item bank (MaRs-IB; Chierchia et al., 2019). Each item in the MaRs-IB consists of a 3 × 3 matrix. Eight of the nine available cells in the matrix are filled with abstract shapes, and one cell in the bottom right-hand corner is left empty. Participants are required to complete the matrix by selecting the missing shape from four possible options. In order to correctly identify the missing shape, participants have to deduce relationships between the shapes in the matrix (which vary in shape, colour, size and position). When participants select an answer, they move on to the next item. If participants do not provide a response within 30 seconds, they continue to the next item without a response. The MaRs-IB assessment lasts eight minutes regardless of how many trials are completed. There is a total of 80 different items in the MaRs-IB, however participants are not required (or expected) to complete all 80 items within the eight minutes. If a participant completed all 80 items within this time limit, the items were presented again but the responses to these were not analysed (following the procedure established by Chierchia et al., 2019). The MaRs-IB has been shown to have acceptable internal consistency (Kuder-Richardson 20 ≥ 0.7) and test–retest reliability (r ≥ 0.7; Chierchia et al., 2019).

### Procedure

**Fig. 1:**
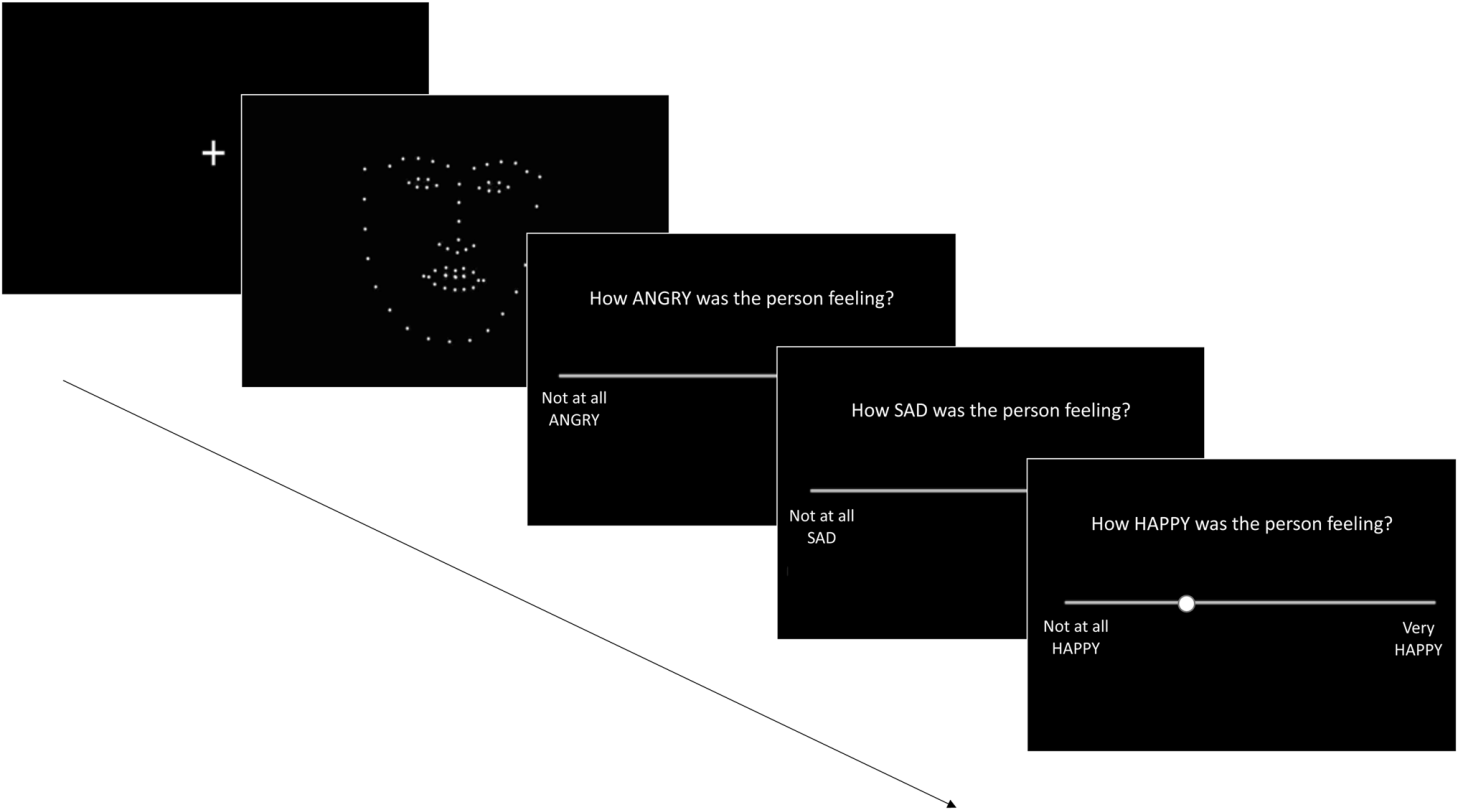
Example of one trial in the PLF task. The fixation cross display is presented for 500 ms at the start of each trial. The average length of a stimulus video was approximately 7 seconds. Rating scales remained on screen until participants had rated the stimulus and pressed the space bar

Following a pre-registered design (see https://osf.io/kpefz), participants first completed the questionnaires (demographics followed by AQ, followed by TAS-20) and then moved on to the PLF task. Each trial in this task began with the presentation of a stimulus, which comprised a silent PLF video of an actor expressing one of 3 emotions, whilst saying a sentence, at one of the 3 spatial and 3 kinematic levels. After watching the video, participants were asked to rate how *angry, happy* and *sad* the person was feeling. Participants made their ratings on a visual analogue scale, with one end representing ‘Not at all *angry/happy/sad*’ and the opposite end representing ‘Very *angry/happy/sad*’. Individuals were asked to make ratings for all three target emotions (angry, happy and sad) on scales, which were presented on screen in a random order, after each PLF video. Each trial took approximately 25 seconds to complete. Participants completed 3 practice trials (at the 100% spatial and 100% speed level) and then 108 randomly ordered experimental trials (12 per condition) across three blocks. Participants were invited to take a break between blocks. The structure of each trial is displayed in Fig. 1. After finishing the PLF task, participants completed the MaRs-IB (Chierchia et al., 2019).

Participants completed all tasks online using Google Chrome or Mozilla Firefox on a computer or laptop. The frame rate (in frames per second; FPS) of their devices was measured to ensure that the quality/fluidity of the stimulus videos was not degraded. All participants’ frame rates were 60 FPS or higher with one exception at 50 FPS. When we ran all analyses with and without the 50 FPS participant, treating them as a potential outlier, the pattern of results was unaffected. Therefore, this participant was included in all analyses.

### Statistical Analysis

The three emotion rating responses for each trial were transformed into magnitude scores from 0 to 10 (with 0 representing a response of ‘Not at all’ and 10 representing ‘Very’) to 3 decimal places. Emotion recognition accuracy scores were calculated as the correct emotion rating minus the mean of the two incorrect emotion ratings.^3^ For instance, for a trial in which an angry PLF was presented, the mean rating of the two incorrect emotions (happy and sad) was subtracted from the rating for the correct emotion (angry).

To test our first hypothesis, we submitted these accuracy scores to a 2 × 3 × 3 × 3 Analysis of Variance (ANOVA) with the between-subjects factor *group* (ASD, control) and the within-subjects factors *emotion* (happy, angry, sad), *stimulus spatial level* (S1, S2, S3), and *stimulus kinematic level* (K1, K2, K3). This analysis has the potential to reveal differences between the groups in their accuracy of emotion recognition from facial motion cues. It is possible, however, that the two groups could have comparable accuracy scores but different patterns of ratings. For example, an accuracy score of 2 for an angry stimulus could relate to an anger magnitude rating of 4 and happy and sad ratings of 2, or an anger rating of 4, happy rating of 0, and a sad rating of 4. To more sensitively pick up on any differences between groups, we also used magnitude as the DV and conducted a 2 × 3 × 3 × 3 × 3 ANOVA with the between subjects factor *group* (ASD, control) and the within-subjects factors *emotion* (happy, angry, sad), *stimulus spatial level* (S1, S2, S3), *stimulus kinematic level* (K1, K2, K3) and *rating* (happy, angry, sad).

To explore whether the effects of the spatial and kinematic manipulation on emotion recognition accuracy covaried with alexithymia scores, we employed multiple regression analyses. More specifically, we applied a square root transformation to all ordinal factors of interest (age, NVR, AQ, TAS-20), computed z-scores for the transformed data, and submitted the transformed z-scored data, along with the nominal predictor *gender,* to multiple regression analyses. The effect of the spatial manipulation (defined as the difference in accuracy between S3 and S1), the effect of the kinematic manipulation (defined as the difference in accuracy between K3 and K1), mean recognition accuracy, and accuracy for angry videos at the normal level (S2, K2) were used as the DVs for each of these analyses. In addition, in order to explore whether autistic and/or alexithymic traits predicted the magnitude of correct and incorrect ratings, we constructed two linear mixed effects models with subject, age, gender and NVR as random intercepts. In these models, ratings for angry facial motion at the normal level, and ratings across all emotions and levels of the spatial and kinematic manipulation, were the DVs respectively. For all analyses, we used a p = 0.05 significance threshold to determine whether to accept or reject the null hypothesis. The frequentist approach was supplemented with the calculation of Bayes Factors, which quantify the relative evidence for one theory or model over another. For all Bayesian analyses, we followed the classification scheme used in JASP (Lee & Wagenmakers, 2014) to classify the strength of evidence given by Bayes factors, with BF_10_ values between one and three considered as weak evidence, between three and ten as moderate evidence and greater than ten as strong evidence for the alternative hypothesis. In addition, BF_10_ values between 1 and 1/3 are considered weak evidence, between 1/3 and 1/10 as moderate evidence, and smaller than 1/10 as strong evidence for the null hypothesis respectively (Lee & Wagenmakers, 2014).

## Results

Our primary hypothesis was that emotion recognition accuracy would be affected by both kinematic and spatial manipulation and that these effects would not interact with group. To test this hypothesis, we conducted a mixed 2 × 3 × 3 × 3 ANOVA with the between-subjects factor *group* (ASD, control) and the within-subjects factors *emotion* (happy, angry, sad), *stimulus spatial level* (S1, S2, S3), and *stimulus kinematic level* (K1, K2, K3). This analysis revealed a significant main effect of emotion [F_(2,116)_ = 17.79, p < 0.001, *η*_*P*_^*2*^ = 0.24, BF_10_ = 1.03e^15^; see Supplementary Information D], a main effect of spatial level [F_(2,116)_ = 259.57, p < 0.001, *η*_*P*_^*2*^ = 0.82, BF_10_ = 9.05e^57^; see Supplementary Information D] which was qualified by an emotion x spatial interaction [F_(4,232)_ = 88.42, p < 0.001, *η*_*P*_^*2*^ = 0.60, BF_10_ = 7.53e^58^], and an emotion × kinematic interaction [F_(4,232)_ = 53.90, p < 0.001, *η*_*P*_^*2*^ = 0.48, BF_10_ = 1.90e^20^]. Furthermore, this analysis revealed a significant four-way emotion × spatial × kinematic × group interaction [F_(8,464)_ = 2.438, p < 0.05, *η*_*P*_^*2*^ = 0.04, BF_10_ = 0.07]. Note that no kinematic × group interaction was found [p = 0.538, BF_10_ = 0.02], suggesting that autistic and control participants exhibit similar patterns of accuracy across the kinematic levels. Below, in order to shed light on the effects of the spatial and kinematic manipulations, we first unpack the emotion × kinematic and emotion × spatial interactions. Subsequently we fully unpack the emotion × spatial × kinematic × group interaction.

**Fig. 2:**
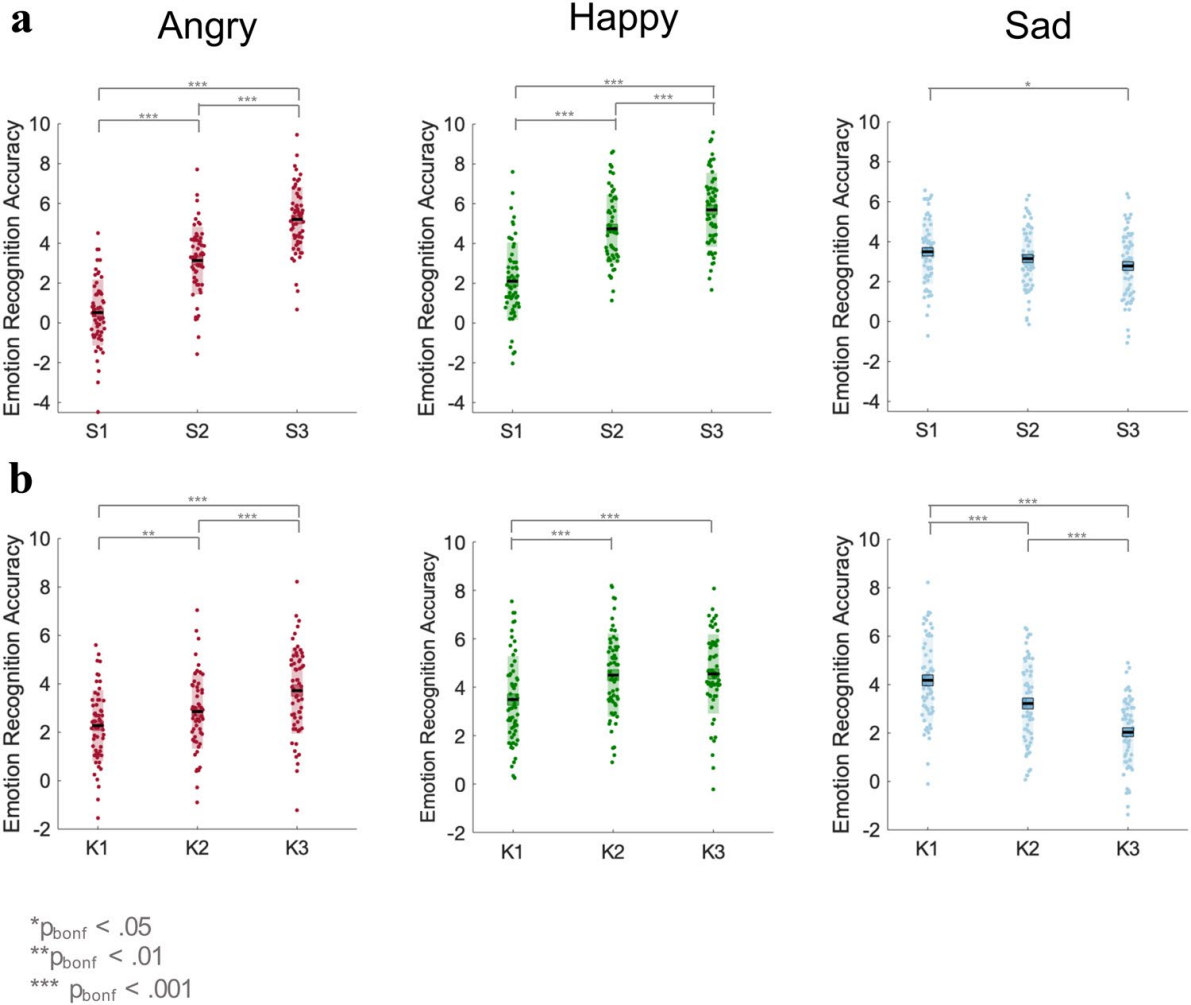
Mean accuracy scores, for all participants, for each emotion across the spatial (panel ** a**) and kinematic (panel **b**) levels. The black line represents the mean, the shaded region represents the standard deviation, the coloured box represents 1 standard error around the mean and the dots are individual data points

In line with Sowden et al., (2021), we observed an emotion × spatial interaction [F_(4,232)_ = 88.42, p < 0.001, *η*_*P*_^*2*^ = 0.60, BF_10_ = 7.53e^58^]. Post-hoc repeated measures ANOVAs revealed that whilst the effect of the spatial manipulation was present for all three emotions (all F > 7.00, all p < 0.01), the direction of the effect varied between high and low arousal emotions: recognition scores for angry and happy facial motion were highest for 150% spatial extent (S3) [angry mean (Standard Error of the Mean; SEM) = 5.21(0.21); happy mean(SEM) = 5.70(0.24)], followed by 100% spatial extent (S2) [angry mean(SEM) = 3.15(0.22); happy mean(SEM) = 4.75(0.23)], and finally 50% spatial extent (S1) [angry mean SEM) = 0.53(0.22); happy mean(SEM) = 2.10(0.25)]. In contrast, for sad facial motion, recognition scores were highest for S1 [sad mean(SEM) = 3.50(0.22)], lowest for S3 [sad mean(SEM) = 2.78(0.22)] and intermediate for S2 [sad mean(SEM) = 3.15(0.20)]. This pattern matches the results reported by Sowden et al., (2021) for non-autistic participants. The emotion recognition accuracy scores for each emotion across the spatial levels can be seen in Fig. 2a.

In addition, our analysis identified an emotion x kinematic interaction [F_(4,232)_ = 53.90, p < 0.001, *η*_*P*_^*2*^ = 0.48, BF_10_ = 1.90e^20^]. Whilst there was a main effect of the kinematic manipulation for all three emotions (all F > 20, all p < 0.001), the direction of the effect differed between high and low arousal emotions. For angry and happy facial motion, emotion recognition improved with increasing speed [angry: K1 mean(SEM) = 2.28(0.19); K2 mean(SEM) = 2.87(0.19); K3 mean(SEM) = 3.73(0.23); happy: K1 mean(SEM) = 3.50(0.23); K2 mean(SEM) = 4.50(0.22); K3 mean(SEM) = 4.55(0.21)]. For sad facial motion, emotion recognition improved as speed decreased [K3 mean(SEM) = 2.03(0.19); K2 mean(SEM) = 3.21(0.22); K1 mean(SEM) = 4.18(0.23)]. This pattern of results also matches the findings from Sowden et al., (2021).^4^ The emotion recognition accuracy scores for each emotion across the kinematic levels can be seen in Fig. 2b.

**Fig. 3:**
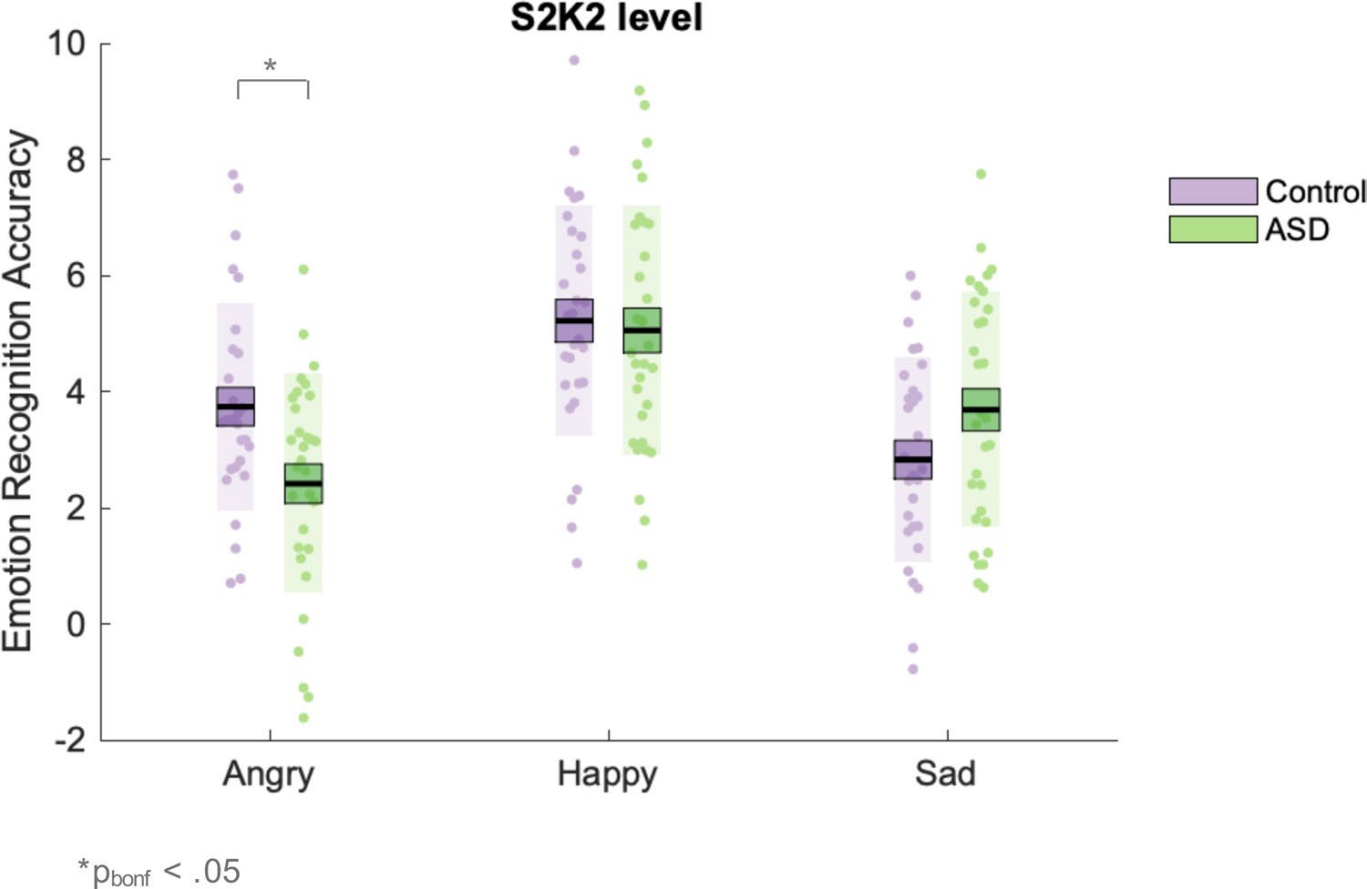
Accuracy at the unmanipulated S2, K2 level, as a function of emotion. Control in lilac, ASD in green. The black line represents the mean, the coloured box represents the standard error of the mean, the shaded region represents the standard deviation, and the dots are individual data points

In order to unpack the significant four-way interaction, we conducted post-hoc 2 × 3 × 3 (group, emotion, kinematic) ANOVAs for each spatial level. This analysis revealed a significant emotion x kinematic x group interaction at the S2 [F_(4,232)_ = 4.53, p < 0.01, *η*_*P*_^*2*^ = 0.07, BF_10_ = 5.92] but not S1 [p = 0.265, BF_10_ = 0.09] or S3 [p = 0.208, BF_10_ = 0.09] level. To unpack this emotion x kinematic x group interaction at the S2 level, we conducted separate post-hoc ANOVAs for each kinematic level at the 100% (S2) spatial level. This analysis revealed a significant emotion x group interaction at the K2 [F_(2,116)_ = 6.48, p < 0.01, η_P_^2^ = 0.10, BF_10_ = 17.09] but not K1 [p = 0.244, BF_10_ = 0.32] or K3 [p = 0.082, BF_10_ = 0.82] level. Bonferroni-corrected post-hoc independent sample t tests revealed that control, relative to ASD, participants had higher accuracy for angry facial motion at the 100% spatial (S2) and speed (K2) level [t(58) = 2.78, p_bonf._ < 0.05, mean difference = 1.48, BF_10_ = 6.09]. There were no significant group differences in emotion recognition accuracy for happy [p = 0.757, BF_10_ = 0.27] or sad [p = 0.085, BF_10_ = 0.93] videos at the S2K2 level. Thus, the groups significantly differed in accuracy for angry PLFs that were not spatially or kinematically manipulated. The mean emotion recognition accuracy scores across each emotion for control and ASD participants at the S2K2 level are shown in Fig. 3.

**Fig. 4:**
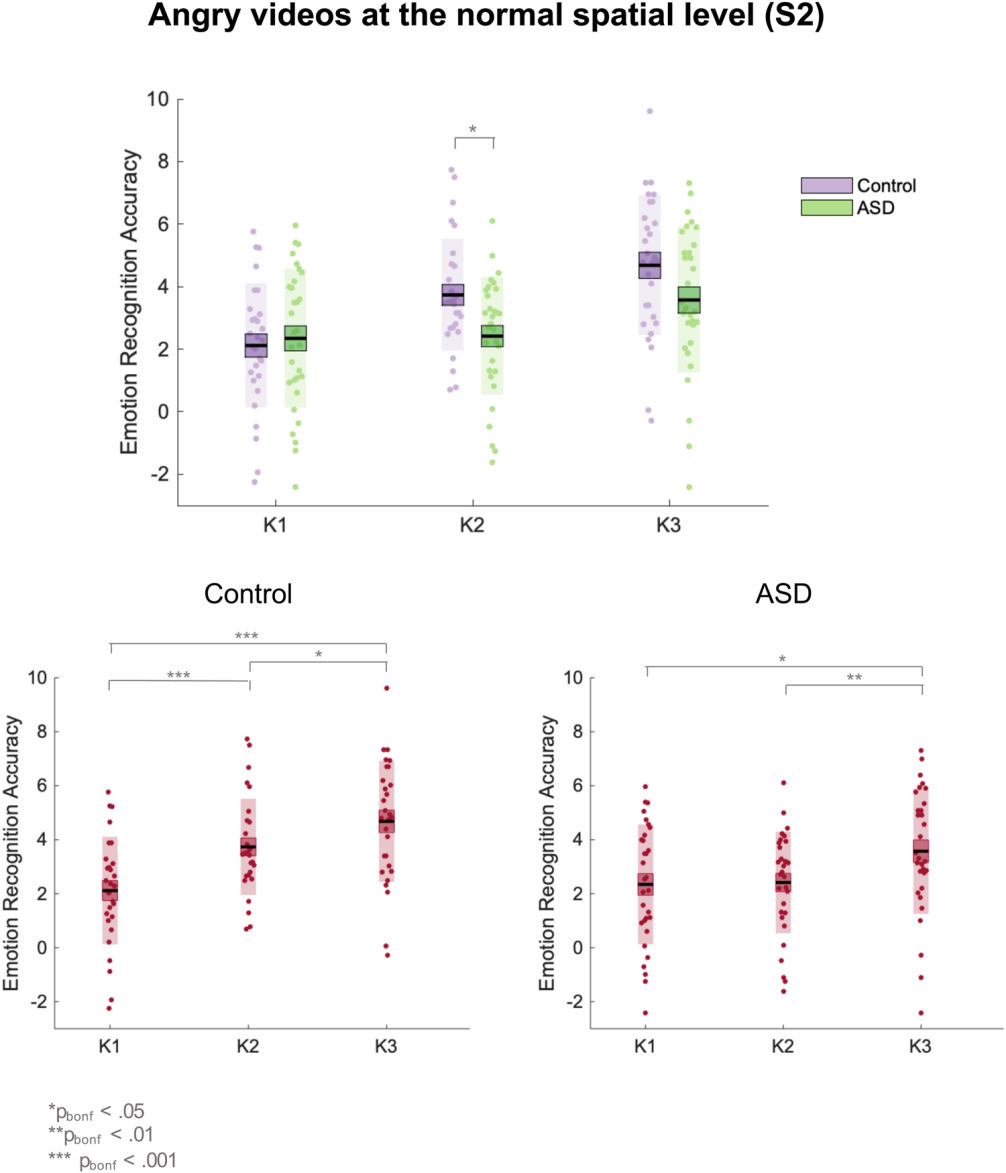
Mean accuracy scores for angry videos at the 100% spatial (S2) level for control and ASD participants across the kinematic levels. The black line represents the mean, the coloured box represents the standard error of the mean, the shaded region represents the standard deviation, and the dots are individual data points

To further unpack the emotion x kinematic x group interaction at the S2 level, we conducted separate post-hoc ANOVAs for each emotion at the S2 level. This analysis identified a significant kinematic × group interaction for angry [F_(2,116)_ = 4.59, p < 0.05, *η*_*P*_^*2*^ = 0.07, BF_10_ = 3.49] but not happy [p = 0.070, BF_10_ = 0.95] or sad [p = 0.123, BF_10_ = 0.53] PLFs. Therefore, for angry videos at the normal spatial level, the effect of the kinematic manipulation varied as a function of group. Bonferroni-corrected paired sample t tests demonstrated that whilst the control group exhibited increasing accuracy across all kinematic levels [K1–K2: t(28) = − 4.31, p_bonf_ < 0.001, mean difference = − 1.62, BF_10_ = 153.77; K2–K3: t(28) =  − 2.86, p_bonf_ < 0.05, mean difference = − 0.95, BF_10_ = 5.52], the ASD group only showed improvement from K2 to K3 [t(30) =  − 3.46, p_bonf_ < 0.01, mean difference = − 1.16, BF_10_ = 21.10] and not K1 to K2 [p = 0.865, BF_10_ = 0.19]. Furthermore, the groups did not significantly differ at K1 (F_(1,58)_ = 0.18, p > 0.05) or K3 (F_(__1,58)_ = 3.53 p > 0.05) but at K2, controls out-performed autistic participants (F_(1,58)_ = 7.75, p < 0.01, *η*_*P*_^*2*^ = 0.12). These results suggest that, whilst controls improved in their accuracy for angry facial motion across each level of increasing kinematic manipulation, for autistic participants, only the most extreme (K3) level of the kinematic manipulation resulted in an accuracy boost. The mean accuracy scores for angry videos across the kinematic levels (at the unmanipulated S2 level) for control and ASD participants are shown in Fig. 4.

In order to compare the magnitude of the ratings between groups, we conducted a mixed 2 × 3 × 3 × 3 × 3 ANOVA with the between subjects factor *group* (ASD, control) and the within-subjects factors *emotion* (happy, angry, sad), *stimulus spatial level* (S1, S2, S3), *stimulus kinematic level* (K1, K2, K3) and *rating* (happy, angry, sad). This analysis revealed a significant main effect of emotion [F_(2, 116)_ = 34.86, p < 0.001, *η*_*P*_^*2*^ = 0.38], spatial level [F_(2,116)_ = 50.52, p < 0.001, *η*_*P*_^*2*^ = 0.47], kinematic level [F_(2,116)_ = 3.51, p < 0.05, *η*_*P*_^*2*^ = 0.06] and rating [F_(2,116)_ = 3.592, p < 0.05, *η*_*P*_^*2*^ = 0.06], as well as emotion × rating [F_(4,232)_ = 489.95, p < 0.001, *η*_*P*_^*2*^ = 0.89], spatial x rating [F_(4,232)_ = 64.26, p < 0.001, *η*_*P*_^*2*^ = 0.53], kinematic × rating [F_(4,232)_ = 49.08, p < 0.001, *η*_*P*_^*2*^ = 0.46], emotion × spatial × rating [F_(8,464)_ = 111.13, p < 0.001, *η*_*P*_^*2*^ = 0.66], emotion × kinematic × rating [F_(8,464)_ = 12.02, p < 0.001, *η*_*P*_^*2*^ = 0.17], kinematic × rating × group [F_(4,232)_ = 2.79, p < 0.05, η_P_^2^ = 0.05] and spatial × kinematic × rating × group [F_(8,464)_ = 2.76, p < 0.05, η_P_^2^ = 0.05] interactions. All these interactions and main effects are unpacked in Supplementary Information F.

**Fig. 5:**
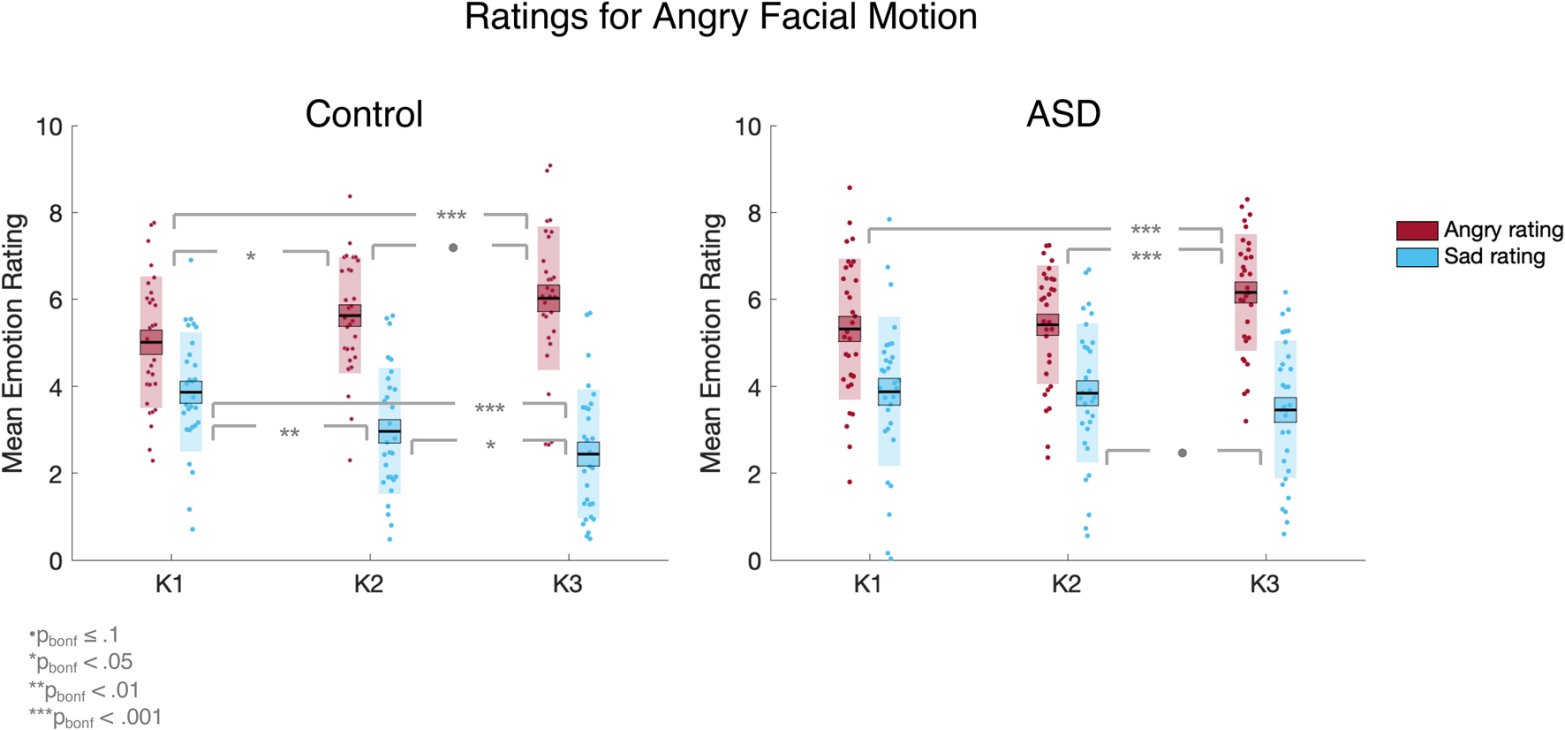
Mean angry and sad ratings given by control and ASD participants for angry facial motion across the kinematic levels. The black line represents the mean, the coloured box represents the standard error of the mean, the shaded region represents the standard deviation, and the dots are individual data points

In addition, this analysis revealed an emotion x kinematic x rating x group interaction which approached significance [F_(8,464)_ = 1.90, p = 0.058, η_P_^2^ = 0.03]. Since this interaction potentially offers further insight about the between group difference in anger recognition, we unpack it in full here. Post-hoc 2 × 3 × 3 ANOVAs (group × kinematic × rating) for each of the emotional videos revealed a significant kinematic x rating x group interaction for angry [F_(4,232)_ = 4.26, p < 0.01, η_P_^2^ = 0.07, BF_10_ = 0.61] but not happy [p = 0.687, BF_10_ = 0.03] or sad [p = 0.122, BF_10_ = 0.09] facial motion. Importantly, post-hoc ANOVAs revealed that for control participants, speeding up angry facial motion (regardless of the spatial level) improves accuracy by increasing ratings of anger [F_(2,56)_ = 15.39, p < 0.001, η_P_^2^ = 0.36, BF_10_ = 3344.71] and lowering ratings of sadness [F_(2,56)_ = 24.15, p < 0.001, η_P_^2^ = 0.46, BF_10_ = 374,155.73] across *all* levels of the kinematic manipulation [angry ratings K1–K2: t(28) = -3.17, p < 0.01, mean difference = -0.62, BF_10_ = 10.71; angry ratings K2–K3: t(28) = -2.24, p < 0.05, mean difference = -0.40, BF_10_ = 1.67; sad ratings K1–K2: t(28) = 3.91, p = 0.001, mean difference = 0.90, BF_10_ = 58.34; sad ratings K2–K3 t(28) = 2.74, p < 0.05, mean difference = 0.52, BF_10_ = 4.34] (however, note that after Bonferroni-correction, the difference in angry ratings for angry facial motion between K2 and K3 became non-significant; p = 0.100; see Fig. 5).

For autistic participants, speeding up angry facial motion also improved accuracy by increasing ratings of anger [F_(2,60)_ = 12.18, p < 0.001, η_P_^2^ = 0.29, BF_10_ = 551.72], however this effect was driven by an increase from the 100% to 150% level [t(30) = -5.24, p = 0.001, mean difference = -0.75, BF_10_ = 1792.14], and not the 50% to 100% level [p = 0.636, BF_10_ = 0.21]. In addition, we found that there was a main effect of kinematic level for sad ratings that approached significance [F_(2,60)_ = 2.89, p = 0.063, η_P_^2^ = 0.09,, BF_10_ = 0.90]. Importantly, sad ratings only decreased from 100% to 150% speed [t(30) = 2.32, p < 0.05, mean difference = 0.39, BF_10_ = 1.94] and not from 50% to 100% speed [p = 0.877, BF_10_ = 0.19] (however, note that after Bonferroni-correction, the difference in sad ratings for angry facial motion between K2 and K3 became non-significant; p = 0.081; see Fig. 5). Consequently, we primarily observe differences in the accuracy of anger recognition between our ASD and control groups because, for the ASD group, speeding up angry facial motion only reduces confusion between angry and sad ratings when the speed is increased from 100% to 150% (and not 50% to 100%). In contrast, for the control group increasing the speed of angry facial motion from 50% to 100% *and* from 100% to 150% reduces confusion between anger and sadness ratings.

### Multiple Regression Analyses

In addition, we aimed to explore whether variation in emotion recognition accuracy covaried with scores on our self-report alexithymia measure (TAS-20). To test whether autistic or alexithymic traits were predictive of the effect of the spatial and kinematic manipulations, we conducted two multiple regression analyses. For the first analysis, we used the effect of spatial manipulation (defined as the difference in accuracy between S3 and S1) as the dependent variable (DV) and AQ and TAS-20 as predictor variables. This analysis resulted in a non-significant model overall [F_(2,57)_ = 0.87, p = 0.425], neither AQ [standardized β = -0.17, t(57) = -1.10, p = 0.274] nor TAS-20 [standardized β = 0.19, t(57) = 1.20, p = 0.236] were significant predictors of the effect of the spatial manipulation. In the second analysis, we used the effect of the kinematic manipulation (defined as the difference in accuracy between K3 and K1) as the DV and AQ and TAS-20 as predictors. Again, this analysis resulted in a non-significant model [F_(2,57)_ = 1.63, p = 0.206], neither AQ [standardized β = 0.20, t(57) = 1.33, p = 0.189] nor TAS-20 [standardized β = 0.05, t(57) = 0.32 p = 0.752] were significant predictors of the effect of the kinematic manipulation. We then conducted a third multiple regression with mean emotion recognition accuracy (across all trials) as the DV. Once again, neither AQ [standardized β = -−.19, t(57) = -−1.24, p = 0.220] nor TAS-20 [standardized β = 0.12, t(57) = 0.81, p = 0.424] were significant predictors of mean recognition accuracy and the overall model did not explain a significant amount of variance in the data [F_(2,57)_ = 0.78, p = 0.461]. To explore the possibility that only extreme scores on the TAS-20 predict performance, we compared mean accuracy for alexithymic (i.e., TAS-20 ≥ 61) and non-alexithymic (i.e., TAS-20 ≤ 51) participants (according to the cut-off scores outlined by Bagby et al., 1994), excluding ‘possibly alexithymic’ individuals. An independent samples t test confirmed that there was no significant difference in mean accuracy between these groups [t(48) = -0.18, p = 0.861, mean difference = -0.05, BF_10_ = 0.29].

Finally, building on our previous observation that the ASD and control groups differed in accuracy for angry facial motion at the normal (100%) spatial and speed level, we conducted a multiple regression analysis to identify the extent to which autistic and alexithymic traits were predictive of accuracy for angry videos at this level. This analysis revealed that autistic [standardized β = -−0.44, t(57) = -−3.05, p < 0.01], but not alexithymic [standardized β = 0.22, t(57) = 1.54, p = 0.130], traits were predictive of accuracy for angry facial motion at the normal spatial and speed level [overall model statistics: F_(2,57)_ = 4.67, p < 0.05, R^2^ = 0.141]. Bayesian analyses revealed that AQ [BF_inclusion_ = 4.230] was over 16 times more likely to be included in a model to predict accuracy for angry videos at the normal spatial and speed level than alexithymic traits [BF_inclusion_ = 0.263].

In order to ensure that AQ is not just a significant predictor of accuracy for angry expressions at the normal spatial and speed level due to variation across other co-variables (e.g., age, gender, and non-verbal reasoning), we completed an additional three-step forced entry hierarchical regression analysis following the procedures of Cook et al., (2013). In the first step, the demographic variables (gender, age and NVR) were entered into the model, which overall accounted for 16% of the variance in accuracy at the S2K2 level [F_(3,56)_ = 3.56, p < 0.05, R^2^ = 0.160]. Importantly, of the three demographic variables, only NVR was a significant predictor of accuracy for angry facial motion at the normal spatial and speed level [standardized β = 0.35, t(56) = 2.79, p < 0.01] (and not gender [standardized β = 0.15, t(56) = 1.20, p = 0.233] or age [standardized β = -−0.01, t(56) = -−0.06, p = 0.950]). In the second step, AQ was added [standardized β = -−0.36, t(55) = -−3,13, p < 0.01], producing a statistically significant R^2^ change [F change_(1, 55)_ = 9.80, p < 0.01, R^2^ change = 0.127]. Finally, when TAS-20 was entered into the model, the analysis revealed it was not a significant predictor of accuracy for angry facial motion at the normal level [standardized β = 0.17, t(54) = 1.26, p = 0.214] and resulted in a non-significant R^2^ change [F change_(1, 54)_ = 1.58, p = 0.214, R^2^ change = 0.020; see Table 2.]. Hence, this analysis demonstrated that autistic traits (and not alexithymic traits) were a significant predictor of accuracy for angry facial motion at the normal level (S2, K2) even after age, gender and NVR have been accounted for.

**Table 2:** Results of the forced entry hierarchical regression for accuracy for angry videos at the normal spatial and speed level

Model	R	R^2^	Adjusted R^2^	SEE	R^2^ change	F change	Sig. F change
1	0.400	0.160	0.115	1.82	0.160	3.556	0.020
2	0.536	0.287	0.235	1.69	0.127	9.798	0.003
3	0.554	0.307	0.243	1.68	0.020	1.581	0.214

*1* Predictors: age, gender, non-verbal reasoning; *2* predictors: age, gender, non-verbal reasoning, AQ; *3* predictors: age, gender, non-verbal reasoning, AQ, TAS-20

These analyses suggest that alexithymia accounts for very little variance in accuracy for angry facial motion at the normal (S2K2) level once autistic traits have been accounted for. However, since our autism and alexithymia measures were correlated [R = 0.53, p < 0.001], when alexithymia is entered into a multiple regression after autistic traits, it may not be a significant predictor due to multi-collinearity. Consequently, we ran one further hierarchical regression, with the demographic variables entered in Step 1, alexithymia in Step 2 and autistic traits in Step 3. Alexithymia failed to significantly improve the model [F change_(1, 55)_ = 0.31, p = 0.581, R^2^ change = 0.005], explaining only 0.5% more variance than that explained by the demographic variables alone. Despite being highly correlated with alexithymia, autistic traits were again a significant predictor of accuracy for angry facial motion at the normal level [standardized β = -0.45, t(54) = -3.33, p < 0.01] when added to the model in Step 3. Adding autistic traits at this step produced a statistically significant R^2^ change [F change_(1, 54)_ = 11.12, p < 0.01, R^2^ change = 0.143], explaining an additional 14.3% of the variance in accuracy.

The above results demonstrate that, compared to NVR, age, gender and alexithymia, autistic traits account for an additional 14.3% of the variance in the accuracy of anger recognition from motion cues at the normal (S2K2) level. In principle, autistic traits might contribute to anger recognition by modulating the magnitude of correct ratings (wherein lower AQ should be related to higher anger ratings for angry stimuli), the magnitude of incorrect ratings (wherein lower AQ should be related to lower happy and sad ratings for angry stimuli), or both. In addition, it is possible that alexithymic traits might contribute to correct and incorrect emotion ratings, but not emotion recognition accuracy (e.g., by contributing to both increased correct and incorrect emotion ratings). To explore these possibilities, and thereby shed light on the psychological mechanisms by which AQ negatively predicts anger recognition, we constructed a linear mixed effects model, predicting the magnitude of ratings with AQ score, TAS-20 score, the interaction between AQ score and rating type (correct vs. incorrect), and the interaction between TAS-20 and rating type (correct vs. incorrect). This analysis revealed a significant AQ × rating type interaction [t(180) = 2.12, p < 0.05], wherein AQ predicted incorrect [t(59.89) = 3.36, p < 0.01] but not correct [p = 0.381] emotion ratings for angry facial motion at the normal level; those with higher AQ scores gave higher incorrect emotion ratings (i.e., happy and sad) for angry facial motion at the normal level. Our analysis also identified that the relationship between TAS-20 and ratings (when averaging across correct and incorrect emotions) for angry facial motion at the normal level approached significance [t(180) = 1.80, p = 0.074]. Note that no TAS × rating type interaction was identified [p = 0.288].

The analyses reported above suggest that autistic traits contribute to anger recognition by modulating the magnitude of incorrect ratings, but not correct, ratings. In addition, these analyses revealed an interesting additional finding: alexithymic traits may be positively predictive of *both* correct and incorrect emotion ratings. Since the analyses reported above were restricted to the normal (S2K2) level for angry facial motion, next, we constructed one further linear mixed effects model (following the procedures outlined above) to investigate whether autistic and/or alexithymic traits are predictive of higher correct and incorrect emotion ratings across all emotions and levels of the spatial and kinematic manipulation. This analysis revealed that TAS-20 score was a significant positive predictor of the magnitude of ratings [t(57.84) = 2.95, p < 0.01], with those higher in alexithymia giving higher intensity (correct and incorrect) ratings across all emotions and levels of the spatial and kinematic manipulation. Importantly, the TAS × rating type interaction was not significant [p = 0.125], suggesting that alexithymic traits were predictive of *both* correct and incorrect emotion ratings. Our analysis also revealed that there was a significant AQ × rating type interaction [t(4800.41) = 2.37, p < 0.05]. In line with our previous analysis, AQ predicted incorrect [t(49.02) = 2.24, p < 0.05] but not correct [p = 0.175] emotion ratings, such that those higher in autistic traits gave higher incorrect ratings.

Therefore, our results suggest that whilst the level of autistic traits is predictive of accuracy for angry facial motion at the normal level (by positively predicting incorrect emotion ratings but not correct emotion ratings), alexithymic traits are not predictive of emotion recognition accuracy across emotions and manipulations but are positively predictive of *both* correct and incorrect emotion ratings.

## Discussion

The current study tested whether autistic individuals, relative to alexithymia-matched controls, have greater difficulty recognising emotions from facial motion cues. We hypothesized that emotion recognition would vary as a function of kinematic and spatial manipulation and that these effects would not interact with diagnostic group, but rather Bayesian statistics would provide evidence that the groups perform comparably. We also aimed to explore whether the effects of spatial and kinematic manipulation on emotion recognition accuracy would covary with scores on a self-report alexithymia measure. In replication of Sowden et al., (2021), our results indicated that emotion recognition accuracy was affected by both spatial and kinematic manipulation. In addition, we identified that emotion recognition accuracy did not covary with alexithymia scores. In conflict with our hypothesis, we observed a significant emotion x spatial x kinematic x group interaction. Further unpacking this interaction revealed that autistic, relative to control, adults showed reduced recognition of angry facial motion at the normal (100%) spatial (S2) and speed (K2) level. Furthermore, whilst control participants improved in accuracy across all kinematic levels, autistic participants only benefitted from the speed increase from the normal (100%) to increased (150%) speed level. Exploration of the magnitude of ratings further demonstrated that, for non-autistic participants, speeding up angry PLFs improved accuracy through a combination of increasing anger ratings and decreasing sad ratings for both the 50–100% *and* 100–150% increase. In contrast, for autistic participants speeding up angry facial motion only increased anger ratings and decreased sad ratings between the 100% and 150% levels (not from 50–100%). In addition, multiple regression analyses revealed that autistic traits and NVR, but not age, gender or alexithymia, were significant predictors of recognition accuracy for angry facial motion at the normal spatial and speed level (where level of autistic traits was a negative predictor and NVR was a positive predictor). Although alexithymic traits were not associated with accuracy, they were associated with higher ratings for *both* the correct and incorrect emotions. Importantly, our results demonstrate that when autistic and control individuals are matched in terms of alexithymia there *are* group differences in recognition accuracy, though these are restricted to angry (not happy or sad) facial motion.

Of particular note is our finding that differences between autistic and control individuals are restricted to the recognition of anger from facial motion. This finding is in line with previous research suggesting that angry expressions are better recognized by non-autistic compared to autistic individuals (Ashwin et al., 2006; Bal et al., 2010; Brewer et al., 2016; Leung et al., 2019; Song & Hakoda, 2018) and is supported by meta-analytic evidence demonstrating greater differences between ASD and control groups in the recognition of angry compared to happy and sad expressions (Lozier et al., 2014). Importantly, however, some of these previous studies did not measure alexithymia (Ashwin et al., 2006; Bal et al., 2010; Leung et al., 2019; Song & Hakoda, 2018) and in those that did, alexithymic and ASD traits were confounded (Brewer et al., 2016), making it impossible to determine whether differences in anger recognition were attributable to alexithymia or ASD. The present study resolves this ambiguity and suggests that difficulties with recognising angry expressions at the ‘normal’ spatial and speed level are related to autism, not alexithymia.

An important observation is that in the current paradigm both groups performed equally well for slowed angry facial motion, but whilst the controls benefitted from all elevations in speed (i.e., from 50% to 100%, and from 100% to 150% speed), the autistic participants only benefitted from the 100% to 150% speed increase. Our analysis of the magnitude of angry, happy and sad ratings for angry PLFs provided further insight into this effect: for non-autistic participants, speeding up angry PLFs from 50% to 100% *and* 100% to 150% speed improved accuracy through a combination of increasing anger ratings and decreasing sad ratings, thereby reducing the confusion between emotions. For autistic participants, speeding up angry facial motion also increased anger ratings and decreased sad ratings, however, this only happened between the 100% and 150% levels (and not from 50% to 100%). This lack of a change in angry and sad ratings from 50% to 100% speed resulted in the autistic participants displaying significantly lower emotion recognition accuracy for angry facial motion at 100% speed. Further to this, the lack of a decrease in sad ratings may also explain why autistic traits were associated with higher incorrect emotion ratings for angry facial motion at the normal level (as found in our linear mixed effects model).

These findings raise the possibility that autistic individuals may have a higher ‘kinematic threshold’ for perceiving anger from facial motion (i.e., an angry expression has to be moving quite quickly before it actually appears angry or *angrier* to ASD participants). This idea builds upon the findings of a previous study that used static photographic stimuli at varying expressive intensities (constructed by repeatedly morphing a full expression with a neutral expression to result in nine intensity levels for each emotion) to estimate identification thresholds (the intensity at which an expression is identified correctly on two consecutive trials) for autistic and control participants (Song & Hakoda, 2018). The authors found that autistic individuals had significantly higher identification thresholds than controls, meaning that a higher intensity was necessary before an expression appeared angry to ASD participants (Song & Hakoda, 2018). Importantly, this study also found no significant group differences in identification thresholds for happiness or sadness (Song & Hakoda, 2018). Song and Hakoda’s findings suggest that autistic individuals have a different identification threshold for *static* angry expressions. For dynamic facial expressions, it may be that autistic and control individuals have a different *‘kinematic identification threshold’* such that the expression must move more quickly (than would be required for control individuals) before it is identified as angry. Further research is necessary to investigate whether the group difference in recognising angry expressions at the unmanipulated spatial and speed level is underpinned by a difference in kinematic identification thresholds.

Another (non-mutually exclusive) explanation for why the autistic individuals may have particular difficulty recognizing angry expressions relates to movement production. Previous studies have documented differences between autistic and control participants in the production of facial expressions of emotion (Brewer et al., 2016; Keating & Cook, 2020). In our study, we used PLF videos that were created by filming four *non-autistic participants* posing different emotional states. Given that autistic and non-autistic individuals produce different facial expressions and that one’s own movement patterns influence the perception and interpretation of the movements of others (Cook, 2016; Eddy & Cook, 2018; Edey et al., 2017; Happé et al., 2017) our autistic participants might have struggled to read emotion in our PLF videos because the expressions were dissimilar to expressions that they would adopt themselves. To date, studies that have documented differences between autistic and control participants in the production of facial expressions of emotion have used non-autistic observer ratings as a measure of the quality of facial expression (i.e., from the perspective of a non-autistic rater, autistic individuals produce expressions which appear “atypical”). Consequently, research has not yet identified what specifically is different about autistic and non-autistic facial expressions. Importantly, differences might be found in the final arrangement of facial features (i.e., spatial differences) or the speed/acceleration/jerk with which individuals reach these expressions (i.e., kinematic differences). Further research is necessary to (i) characterize the expressive differences between autistic and non-autistic individuals, (ii) ascertain whether there are greater expressive differences between the groups for angry compared to happy and sad expressions and, (iii) confirm whether such differences in movement profile contribute to emotion recognition difficulties.

There is growing support for the alexithymia hypothesis, not only with respect to facial emotion recognition (e.g., Cook et al., 2013; Milosavljevic et al., 2016; Oakley et al., 2016; Ola & Gullon-Scott, 2020), but also with vocal and musical emotion recognition (Allen et al., 2013; Heaton et al., 2012), and in related domains such as empathy (Bird et al., 2010). As these literatures grow, establishing what can and cannot be explained by the alexithymia hypothesis is of increasing importance not only to academics working in the field but also to clinicians for whom it is important to understand which aspects of behaviour and cognition are indicative of autism, and which are more representative of alexithymia. In the present study, we found that self-reported alexithymia was not predictive of the effect of spatial or kinematic manipulation on emotion recognition from motion cues, emotion recognition accuracy in general, or emotion recognition accuracy specifically relating to angry videos at the normal spatial and speed level. However, when we decomposed our accuracy measure into the magnitude of ratings for the correct and incorrect emotions, we found that elevated alexithymia was associated with increased ratings for both correct and incorrect emotions. Consequently, these data suggest that, in the context of our task, individuals with high levels of alexithymic traits can recognise emotion from motion cues to the extent that they can, for example, rate an angry PLF as *more* angry, relative to happy and sad. However, compared to individuals low in alexithymic traits, they are more likely to rate a PLF high for *all* emotion categories.

One possible explanation for the absence of a significant relationship between alexithymia and emotion recognition accuracy in our study is linked to the use of degraded facial motion stimuli. Bird et al. (2011) demonstrated that impairments in emotion recognition in highly alexithymic individuals may be driven by an avoidance of the eye region. It is possible that, by using degraded stimuli in which the eye-region is represented by the kinematics and spatial configuration of only 6 landmarks (white dots), we have changed the way in which attention is allocated across the face. We know, from previous work, that the speed of movement of our eye-region landmarks carries emotion-differentiating signals (Sowden et al, 2021). However, it is possible that when eyes are represented as six white dots, they are no longer avoided by highly alexithymic individuals. Thus, alexithymic individuals might process information from the eye-region of our PLF stimuli more than they would with, for example, photographic stimuli. It is also conceivable that our PLF stimuli encourage (all) observers’ attention towards the mouth over the eye region. If this were the case, a correlation between alexithymia and impaired emotion recognition may be hidden since there is no known link between alexithymia and impaired recognition of emotion from mouth-region cues.

Perhaps of most interest for the field of alexithymia research is our finding that alexithymic traits are predictive of increased magnitude of *both* correct and incorrect emotion ratings. Such results are reminiscent of a literature which concerns increased emotional reactivity in alexithymic individuals (Lyvers et al., 2018). However, whilst it is tempting to speculate that our results are indicative of *over-*attribution of emotion in highly alexithymic individuals, it should be noted that there is no objective ground-truth with respect to the magnitude of ratings of our PLF stimuli. Our stimuli were designed to discretely represent happy, angry and sad emotions. Therefore, one may argue that the “ground-truth” for an angry PLF, for example, is that happy and sad ratings should be zero. However, we cannot guarantee that our PLF actors did not inadvertently produce mixed emotional expressions. A broader point here is that, given the paucity of research concerning emotion-related facial motion cues, the extent to which facial movements overlap between happy, angry and sad expressions is currently unclear. Thus, whilst it may be that highly alexithymic individuals are *over-attributing* emotion, an alternative possibility is that they are more finely tuned to emotion-related motion cues and are in fact correctly identifying that some motion cues are linked to happy, sad and angry states (though perhaps with different probabilities). To resolve this interpretational issue, further research is required to establish the extent of overlap between dynamic happy, angry and sad expressions.

### Limitations

In the present study, we aimed to produce statistically rigorous and replicable results. The standard alpha level (p < 0.05) has recently been called into question for its utility and appropriateness in psychological research (Amrhein & Greenland, 2018; Benjamin et al., 2018; Halsey et al., 2015; Lakens et al., 2018). Hence, we are reassured to see that our main findings remain significant, after Bonferroni-correction and, when we set a more conservative alpha threshold of 0.025. Importantly, substantial effect sizes and Bayes factors support our low p values, thus providing us with further confidence in our results. Therefore, we believe our findings make sound contributions to the literatures regarding alexithymia, ASD and dynamic facial expression recognition, however, there are several limitations to consider.

One potential limitation is that due to COVID-19-related restrictions on face-to-face testing, only 22 of our ASD group completed ADOS-2 assessments. As a result, we have limited information about whether the remaining 9 participants would surpass the threshold for an autism or autism spectrum diagnosis on the ADOS-2. In addition, of the 22 participants that did complete the observational assessment, just 16 met criteria for a diagnosis. Hence, it is possible that our ASD group display less frequent or lower intensity autistic behaviours than would typically be seen in an ASD population. In spite of this we identified a significant group difference. Note that this limitation may have resulted in false negatives or an underestimation of the true effect size. However, it is highly unlikely that it could have resulted in false positives or inflated effects sizes.

Another potential limitation of this study is that we used the self-report TAS-20 to measure alexithymia. Whilst 89% of studies comparing the emotional self-awareness of autistic and non-autistic participants use self-report measures (and 62% use the TAS-20; Huggins et al., 2020), some authors (e.g., Leising et al., 2009; Marchesi et al., 2014) have questioned their utility as “people with alexithymia, by definition, should not be able to report their psychological state” (Marchesi et al., 2014). However, endeavours to develop objective measures of alexithymia are in their infancy and early attempts are yet to be replicated (e.g., Gaigg et al., 2018; Hickman et al., 2021) and thus self-report measures are necessary. Whilst the TAS-20 has long been the gold-standard tool for assessing alexithymia, there are some concerns that it might actually be a measure of psychopathology symptoms or current levels of psychological distress (see Badura, 2003; Helmes et al., 2008; Leising et al., 2009; Marchesi et al., 2014; Preece et al., 2020; Rief et al., 1996). Further studies may try to replicate our results using alternative measures of alexithymia such as the Perth Alexithymia Questionnaire (Preece et al., 2018) or Bermond Vorst Alexithymia Questionnaire (BVAQ; Vorst & Bermond, 2001), which have been argued to index an alexithymia construct that is distinct from individuals’ current level of psychological distress (Preece et al., 2020). However, since our aim was to investigate whether the alexithymia hypothesis applies, not only to emotion recognition from static face stimuli, but also to recognition from dynamic stimuli, it was crucial that we employ the same measure of alexithymia (i.e., the TAS-20) as has previously been used in the emotion recognition literature (Cook et al., 2013; Milosavljevic et al., 2016; Oakley et al., 2016; Ola & Gullon-Scott, 2020).

Finally, the results of the current study are informative with respect to the recognition of emotion from facial motion cues. However, given that surface properties (Sormaz et al., 2016), such as pigmentation/colouring (Yasuda, 2007) and shading/depth (Wang et al., 2017), are implicated in the recognition of emotion, one should be cautious about assuming that our findings generalise to full dynamic emotional expressions (e.g., video stimuli). Future research should aim to clarify whether our findings are specific to the recognition of emotion from facial motion cues, or if they are applicable more broadly to emotion recognition from full dynamic displays.

## Conclusions

The current study tested whether autistic, relative to alexithymia-matched controls, have greater difficulty recognising emotions from facial motion cues. In conflict with our hypotheses, we observed that autistic, relative to control, adults showed reduced recognition of angry facial motion at the normal (100%) spatial and speed level. Interestingly, whilst for controls recognition accuracy improved across all levels of the kinematic manipulation for angry videos, autistic participants only benefitted from the 100% to 150% speed increase. Alexithymic traits were associated with elevated correct and elevated incorrect emotion ratings, but not accuracy. Our results draw attention to anger specific differences in emotion recognition between autistic and non-autistic individuals. Future research should aim to elucidate why autistic individuals exhibit differences that are specific to angry expressions.

## Supplementary Information

Below is the link to the electronic supplementary material.

Supplementary file1 (DOCX 1639 kb)

## Footnotes

1 ‘Condition-first’ terminology is used throughout in line with the majority preference expressed in a survey of the autistic community (Kenny et al., 2016).

2 Facial morph stimuli were constructed by successively presenting 26 images from a neutral (0%) to full emotional (100%) expression with an increase of 4% in emotion from one image to the next. By presenting the images in this way, it gave the illusion of a dynamic emotional expression. The speed of playback was then manipulated to allow the researchers to test their hypotheses.

3 Many of the studies that have investigated the emotion recognition ability of autistic individuals have used forced-choice paradigms in which there is a binary (correct; 1, or incorrect; 0) accuracy score for each trial. In order to facilitate comparison of our results to those studies, we also completed a binary accuracy analysis, which yielded similar results (see Supplementary Information C). In this analysis, for each trial, participants scored 1 when they gave the highest rating to the correct emotion, and 0 when they rated either of the incorrect emotions higher than the correct emotion.

4 See Supplementary Information E for a juxtaposition of the current data against data published by Sowden et al. (2021).
