# Supplementary material for: Differences Between Autistic and Non-Autistic Adults in the Recognition of Anger from Facial Motion Remain after Controlling for Alexithymia
Source: J Autism Dev Disord. 2021 May 28;52(4):1855–71. doi: 10.1007/s10803-021-05083-9 (PMC8159724; doi:10.1007/s10803-021-05083-9)
Supplement: Supplementary file 1 — Supplementary file1 (DOCX 1639 kb) [file 10803_2021_5083_MOESM1_ESM.docx]

**Supplementary Information**

**Supplementary Information A- Participant Demographics**

Table 1. Participant written responses to the question 'What is your ethnic group?'.

| **Ethnic group** | **N** | ***%*** |
| --- | --- | --- |
| White English/Welsh/Scottish/Northern Irish/British | 25 | 41.7 |
| White/Caucasian | 8 | 13.3 |
| White Polish | 6 | 10.0 |
| White Portuguese | 2 | 3.3 |
| White Italian | 2 | 3.3 |
| Mixed/Multiple Ethnic Groups- White and Asian | 2 | 3.3 |
| European | 2 | 3.3 |
| Polish | 2 | 3.3 |
| Black African | 1 | 1.7 |
| Mixed/Multiple Ethnic Groups- Other | 1 | 1.7 |
| Asian Pakistani | 1 | 1.7 |
| Asian Indian | 1 | 1.7 |
| White Slavic | 1 | 1.7 |
| White Albanian | 1 | 1.7 |
| Black Caribbean | 1 | 1.7 |
| White Hungarian/Greek | 1 | 1.7 |
| British | 1 | 1.7 |
| Latino/Hispanic | 1 | 1.7 |
| White European | 1 | 1.7 |

**Supplementary Information B- Quantity of autistic participants that met criteria for diagnosis**

The level of autistic characteristics of 22 individuals in the ASD group was assessed using the Autism Diagnostic Observation Schedule (version 2 (ADOS-2; Lord et al., 2012). Of the 22 who completed the ADOS-2 assessments, 16 met ADOS criteria for ASD (7 autism, 9 autism spectrum). Although six individuals in the ASD group did not meet criteria for ASD according to the ADOS, they had previously received diagnoses from independent clinicians, and thus still participated in the study. Unfortunately, it was not feasible to complete observational assessments on all ASD participants due to restrictions on face-to-face testing during the COVID-19 pandemic.

**Supplementary Information C- Binary accuracy analysis**

To facilitate completion of a binary accuracy analysis (i.e. correct; 1 or incorrect; 0), we transformed the data such that participants scored 1 when they gave the highest emotion rating to the correct emotion, and 0 when they rated either of the incorrect emotions higher than the correct emotion. We then multiplied these values by 100 such that the emotion recognition scores would reflect percentage accuracy (i.e. the percentage of trials where participants gave the highest rating to the correct emotion). We then submitted these binary accuracy scores to a 2 x 3 x 3 x 3 Analysis of Variance (ANOVA) with the between-subjects factor *group* (ASD, control) and the within-subjects factors *emotion* (happy, angry, sad), *stimulus spatial level* (S1, S2, S3), and *stimulus kinematic level* (K1, K2, K3).

Mirroring the results reported in the main manuscript, this analysis revealed a significant main effect of emotion [F(2, 116) = 27.05, p < .001, *η_P_^2^* = .32, BF_10_ = 5.66e^21^], with percentage accuracy highest for happy [mean(SEM) = 76.10(1.73)], and comparable for angry [mean(SEM) = 59.76(1.99)] and sad [mean(SEM) = 61.74(1.89)] expressions.

We also identified a main effect of spatial level [F(2,116) = 109.11, p < .001, *η_P_^2^* = .65, BF_10_ = 6.01e^29^], with percentage accuracy lowest at the S1 level [mean(SEM) = 54.14(1.74)] and comparable at the S2 [mean(SEM) = 70.62(1.40)] and S3 [mean(SEM) = 72.84(1.26)] levels. In line with the findings reported in the main manuscript, the effect of spatial level was qualified by an emotion x spatial interaction [F(4,232) = 80.66, p < .001, *η_P_^2^* = .58, BF_10_ = 2.77e^54^]. Post-hoc repeated measures ANOVAs revealed that whilst there was an effect of the spatial manipulation for all three emotions (all F > 18, all p < .001) , the direction of the effect varied between high and low arousal emotions (mirroring the results reported in the main manuscript): percentage accuracy for angry and happy expressions were highest for 150% spatial extent (S3) [angry mean(SEM) = 78.82(1.84); happy mean(SEM) = 85.67(1.78)], followed by 100% spatial extent (S2) [angry mean(SEM) = 66.13(2.30); happy mean(SEM) = 82.97(1.74)], followed by 50% spatial extent (S1) [angry mean(SEM) = 34.32(2.84); happy mean(SEM) = 59.67(3.11)]. In contrast, accuracy improved for sad expressions as the level of the spatial manipulation decreased [S3 mean(SEM) = 54.02(2.33); S2 mean(SEM) = 62.76(2.27); S1 mean(SEM) = 68.44(2.42)]; **Fig. 1**].

In addition, we identified a main effect of kinematic level [F(2,116) = 4.651, p < .05, *η_P_^2^* = .07, BF_10_ = 0.04], with percentage accuracy highest at the K2 level [mean(SEM) = 67.64(1.33)], and comparable at the K3 [mean(SEM) = 65.48(1.37)] and K1 levels [mean(SEM) = 64.48(1.45)]. In line with the results reported in the main manuscript, this main effect of kinematic level was qualified by an emotion x kinematic interaction [F(4,232) = 38. 59, p < .001, *η_P_^2^* = .40, BF_10_ = 4.80e^18^]. Post-hoc repeated measures ANOVAs indicated that whilst there was an effect of the kinematic manipulation across all three emotions (all F > 11, p < .001), the direction of the effect was different between high and low arousal emotions (in line with the results reported in the main manuscript): percentage accuracy for angry and happy expressions were highest for the 150% speed (K3) [angry mean(SEM) = 67.90(2.57); happy mean(SEM) = 78.90(1.84)], followed by 100% speed (K2) [angry mean(SEM) = 60.65(2.09); happy mean(SEM) = 78.30(2.01)], followed by 50% speed (K1) [angry mean(SEM) = 50.73(2.49); happy mean(SEM) = 71.11(2.17)]. In contrast, accuracy for sad expressions improved as speed decreased (K3 mean(SEM) = 49.63(2.17); K2 mean(SEM) = 63.98(2.35); K1 mean(SEM) = 71.59(2.52); **Fig. 1**].

*p_bonf_ < .05

** p_bonf_ < .01

***p_bonf_ < .001

**Fig. 1** Percentage accuracy scores, for all participants, for each emotion across the spatial and kinematic levels. The black line represents the mean, the shaded region represents the standard deviation, the coloured box represents 1 standard error around the mean and the dots are individual datapoints

Finally, this analysis also revealed an emotion x kinematic x group interaction [F(4,232) = 2.74, p < .05, *η_P_^2^* = .05, BF_10_ = 0.13]. In order to unpack this significant emotion x kinematic x group interaction, we conducted post-hoc 2 x 3 (group, emotion) ANOVAs for each kinematic level. This analysis revealed a significant emotion x group interaction at the K2 [F(2,116) = 3.93, p < .05, *η_P_^2^ =* .06, BF_10_ = 2.89] but not K1 [p = .616, BF_10_ = 1.64e^-4^] or K3 [p = .548, BF_10_ = 0.18] levels. Bonferroni-correct post-hoc independent sample t-tests revealed that control, relative to autistic, participants had higher accuracy for angry videos at the 100% speed level (averaged across all spatial levels) [t(58) = 3.30, p_bonf_ < .01, mean difference = 13.77, BF_10_ = 20.06]. There were no significant group differences in percentage accuracy for happy [p = .133, BF_10_ = 0.30] or sad [p = .572, BF_10_ = 0.68] expressions at this 100% speed level (**Fig. 2**).

In order to establish whether any group differences at the S2K2 (100% spatial extent, 100% speed) level (that we found in the accuracy analyses in the main paper) were driving the group difference we identified for angry expressions at the K2 level (when averaged across spatial levels), we conducted a further three Bonferroni-corrected independent sample t-tests for angry expressions at the K2 level, at each of the spatial levels. This revealed that controls had significantly higher emotion recognition accuracy for angry expressions at the S2K2 level [t(58) = 3.61, p_bonf_ < .01, mean difference = 20.33, BF_10_ = 40.59], but not at the S1K2 [p_bonf_ = .231, BF_10_ = 1.00] or S3K2 [p_bonf_ = .072, BF_10_ = 2.42] levels, thus mirroring the results reported in the main manuscript. Hence, our findings suggest that the group difference in accuracy for angry expressions discovered at the K2 level (across the spatial levels) may be mainly driven by a group difference at the S2K2 level.

**Fig. 2** Percentage accuracy at the K2 (100%) speed level, as a function of emotion. Control in lilac, ASD in green. The black line represents the mean, the coloured box represents the standard error of the mean, the shaded region represents the standard deviation, and the dots are individual datapoints

**Supplementary Information D- Unpacking the significant main effects of Emotion and Spatial level**

In our main analysis, we conducted a mixed 2 x 3 x 3 x 3 ANOVA with the between-subjects factor *group* (ASD, control) and the within-subjects factors *emotion* (happy, angry, sad), *stimulus spatial level* (S1, S2, S3), and *stimulus kinematic level* (K1, K2, K3). This analysis revealed a significant main effect of emotion [F(2,116) = 17.79, p < .001, *η_P_^2^* = .24, BF_10_ = 4.83e^13^], with recognition scores highest for happy [mean(SEM) = 4.19(.19)], and comparable for sad [mean(SEM) = 3.14(.18)] and angry [mean(SEM) = 2.96(.18)] videos. The main analysis also revealed a main effect of spatial level [F(2,116) = 259.57, p < .001, *η_P_^2^* = .82, BF_10_ = 7.62e^61^], with recognition scores improving as the spatial level increased [S1 mean(SEM) = 2.04(.13); S2 mean(SEM) = 3.68(.16); S3 mean(SEM) = 4.56(.15)].

**Supplementary Information E- Comparing our findings with Sowden et al., (2021)**

**Fig. 3** A graph to compare the results from Experiment 3 and Experiment 4 in Sowden et al., (2021) and from the present study. The graphs depict the mean accuracy scores for all participants, for each emotion across the kinematic levels. The black line represents the mean, the shaded region represents the standard deviation, the colored box represents 1 standard error around the mean and the dots are individual datapoints

In the present study, we identified that for angry videos, emotion recognition improved with increasing speed [Angry: K1 mean(SEM) = 2.28(.19); K2 mean(SEM) = 2.87(.19); K3 mean(SEM) = 3.73(.23)], thus supporting the results from both ‘Experiment 3’ and ‘Experiment 4’ in Sowden et al., (2021; **Fig. 3**). For happy videos, we identified that recognition accuracy improved from K1 to K2 but didn’t from K2 to K3 [Happy: K1 mean(SEM) = 3.50 (.23); K2 mean(SEM) = 4.50(.22); K3 mean(SEM) = 4.55(.21)]. Hence, these findings contradict the evidence from ‘Experiment 3’ and favor the results from ‘Experiment 4’ in Sowden et al., (2021.). For sad videos, emotion recognition improved as speed decreased [K3 mean(SEM) = 2.03(.19); K2 mean(SEM) = 3.21(.22); K1 mean(SEM) = 4.18(.23)], thus confirming the findings from ‘Experiment 3’ and ‘Experiment 4’ in Sowden et al., (2021).

**Supplementary Information F- Unpacking the significant main effects and interactions in the emotion rating analysis**

**Main effects:**

In order to compare the magnitude of the ratings between groups, we conducted a mixed 2 x 3 x 3 x 3 x 3 ANOVA with the between subjects factor *group* (ASD, control) and the within-subjects factors *emotion* (happy, angry, sad), *stimulus spatial level* (S1, S2, S3), *stimulus kinematic level* (K1, K2, K3) and rating (happy, angry, sad). This analysis revealed a significant main effect of emotion [F(2,116) = 34.86, p < .001, *η_P_^2^* = .38], with ratings being highest for angry [mean(SEM) = 3.61(.12)], intermediate for sad [mean(SEM) = 3.48(.12)] and lowest for happy [mean(SEM) = 3.27(.11)] facial motion.

This analysis also revealed a main effect of spatial level [F(2,116) = 50.52, p < .001, *η_P_^2^* = .47], with participants giving the highest ratings at the S3 [mean(SEM) = 3.70(.10)], followed by the S2 [mean(SEM) = 3.43(.11)], followed by the S1 level [mean(SEM) = 3.22(.13)]. The effect of spatial level was qualified by an emotion x spatial interaction [F(4,232) = 3.48, p < .05, *η_P_^2^* = .06]. Post-hoc repeated measures ANOVAs revealed that the effect of the spatial manipulation was strongest for angry facial motion [F(2,116) = 39.74, p < .001, *η_P_^2^* = .41] followed by sad facial motion [F(2,116) = 35.25, p < .001, *η_P_^2^* = .38], followed by happy facial motion [F(2,116) = 15.75, p < .001, *η_P_^2^* = .21].

The 3 x 3 x 3 x 3 ANOVA also revealed a main effect of kinematic level [F(2,116) = 3.51, p < .05, *η_P_^2^* = .06]: participants gave the highest ratings at the K1 level [mean(SEM) = 3.50(.11)], followed by the K3 [mean(SEM) = 3.44(.11)], followed by the K1 level [mean(SEM) = 3.42(.11)].

This analysis revealed a significant main effect of rating [F(2,116) = 3.592, p < .05, *η_P_^2^* = .06], with participants giving the highest sad ratings [mean(SEM) = 3.65(.13)], and comparable angry [mean(SEM) = 3.37(.15)] and happy ratings [mean(SEM) = 3.33(.13)] (regardless of which emotion was shown in the PLF).

**Interactions:**

This main effect of rating was qualified by an emotion x rating interaction [F(4,232) = 489.95, p < .001, *η_P_^2^* = .89]. Whilst there was a main effect of rating for all three emotions (all F > 179, all p < .001), the direction of the effect differed across all three emotions. As one might expect, for angry facial motion, angry ratings were highest [mean(SEM) = 5.59(.18)], followed by sad ratings [mean(SEM) = 3.41(.18)], followed by happy ratings [mean(SEM) = 1.85(.15)]. For happy facial motion, happy ratings were higher [mean(SEM) = 6.06(.16)] than angry [mean(SEM) = 1.77(.14)] and sad [mean(SEM) = 1.98(.13)] ratings (which were comparably low). Finally, for sad facial motion, sad ratings were highest [mean(SEM) = 5.57(.15)], followed by angry ratings [mean(SEM) = 2.77(.17)] and then happy ratings [mean(SEM) = 2.09(.15)].

In addition, we identified a spatial x rating interaction [F(4,232) = 64.26, p < .001, *η_P_^2^* = .53]. Whilst there was a main effect of spatial for all three ratings (all F > 32, all p < .001), the direction of the effect differed for high and low arousal emotion ratings. Regardless of which emotion was shown, angry and happy ratings were highest at the S3 level [angry mean(SEM) = 4.19(.14); happy mean(SEM) = 3.62(.11)], followed by the S2 level [angry mean(SEM) = 3.33(.14); happy mean (SEM) = 3.48(.13)], followed by the S1 level [angry mean(SEM) = 2.61(.19); happy mean(SEM) = 2.89(.17)]. In contrast, regardless of which emotion was shown, sad ratings were highest at the S1 level [mean(SEM) = 4.17(.16)], intermediate at the S2 level [mean(SEM) = 3.49(.13)] and lowest at the S3 level [mean(SEM) = 3.30(.14)]

Our main analysis also revealed a kinematic x rating interaction [F(4,232) = 49.08, p < .001, *η_P_^2^* = .46]. Whilst there was a main effect of spatial for all three ratings (all F > 11, all p < . 001), the direction of the effect across emotions. Regardless of which emotion was shown: angry ratings were highest at the K3 level [mean(SEM) = 3.74(.15)] and comparable at the K1 [mean(SEM) = 3.15(.17)] and K2 [mean(SEM) = 3.23(.14)] levels; happy ratings were highest at the K3 [mean(SEM) = 3.45(.13)] and K2 [mean(SEM) = 3.40(.13)] levels, and lower at the K1 [mean(SEM) = 3.15(.13)] level; and sad ratings were highest at the K1 [mean(SEM) = 4.20(.14)], followed by the K2 [mean(SEM) = 3.62(.14)], followed by the K3 [mean(SEM) = 3.13(.14)] level.

These interactions were further qualified by an emotion x spatial x rating interaction [F(8,464) = 111.13, p < .001, *η_P_^2^* = .66]. Unpacking this interaction facilitated exploration of which specific emotion confusions were made as the PLF stimulus videos transitioned away from their typical spatial extent. Post-hoc repeated measures ANOVAs indicated that a spatial x rating interaction was present for all emotional videos (all F > 98, p < .001), but that this effect differed across these emotions. As the spatial level of angry facial motion decreased, they were rated as less angry [F(2,116) = 247.43, p < .001, *η_P_^2^* = .81], and were more likely to be confused for happy [F(2,116) = 21.96, p < .001, *η_P_^2^* = .28] and sad [F(2,116) = 23.54, p < .001, *η_P_^2^* = .29]. In addition, as the spatial level of happy facial motion decreased, they were rated as less happy [F(2,116) = 143.11, p < .001, *η_P_^2^* = .71], and were more likely to be confused for sad [F(2,116) = 62.17, p < .001, *η_P_^2^* = .52] but not angry [p = .061]. In contrast, as the spatial level of sad facial motion increased, there were no differences in sad ratings [p = .894,] or happy ratings [p = .256], but they *were* more likely to be confused for angry [F(2,116) = 40.54, p < .001, *η_P_^2^* = .41].

In addition, our main analysis found an emotion x kinematic x rating interaction [F(8,464) = 12.02, p < .001, *η_P_^2^* = .17]. Unpacking this interaction facilitated exploration of which specific emotion confusions were made as the PLF stimulus videos transitioned away from their typical spatial extent. Post-hoc repeated measures ANOVAs indicated that whilst a kinematic x rating interaction was present for all emotional videos (all F > 179, p < .001), but that this effect differed across these emotions. Our analysis identified that as the speed of angry facial motion decreased, they were rated as less angry [F(2,116) = 25.39, p < .001, *η_P_^2^* = .30], and were more likely to be confused for sad [F(2,116) = 21.11, p < .001, *η_P_^2^* = .27]. Note that there were no differences in happy ratings for angry facial motion as speed changes [p = .264]. In addition, we found that as the speed of happy facial motion decreased, they were rated as less happy [F(2,116) = 15.84, p < .001, *η_P_^2^* = .21], and were more likely to be confused for sad [F(2,116) = 33.73, p < .001, *η_P_^2^* = .37]. Note that happy facial motion was more likely to be confused for angry at the K1 and K3 levels than at the K2 level [F(2,116) = 4.37, p < .05, *η_P_^2^* = .07]. Finally, we found that as the speed of sad facial motion increased, they were rated as less sad [F(2,116) = 58.18, p < .001, *η_P_^2^* = .50], and were more likely to be confused for angry [F(2,116) = 30.56, p < .001, *η_P_^2^* = .35] and happy [F(2,116) = 9.54, p < .001, *η_P_^2^* = .14]

In addition, we identified a kinematic x rating x group interaction [F(4,232) = 2.79, p < .05, *η_P_^2^* = .05] and a spatial x kinematic x rating x group interaction [F(8,464) = 2.76, p < .05, *η_P_^2^* = .05]. To unpack the first of these interactions, we conducted post-hoc 2 x 3 ANOVAs (group x kinematic) for each emotion rating. This analysis revealed a significant kinematic x group interaction for sad [F(2,116) = 3.45, p < .05, *η_P_^2^* = .06], but not angry [p = .110] or happy [p = .474] ratings. Whilst there was a significant effect of the kinematic manipulation on sad ratings for both control [F(2,56) = 46.98, p < .001, *η_P_^2^* = .63], and autistic [F(2,60) = 26.85, p < .001, *η_P_^2^* = .47] participants, the effect of the kinematic manipulation was greater for controls (see **Fig. 4**). In other words, regardless of what emotion was displayed, the sad ratings given by autistic (relative to control) participants were less affected by the kinematic manipulation. In our 2 x 3 ANOVA (group x kinematic) for sad ratings, we also identified a main effect of group, with autistic participants giving higher mean sad ratings (regardless of what emotion was displayed) [t(58) = -2.11, p < .05, mean difference = -0.56].


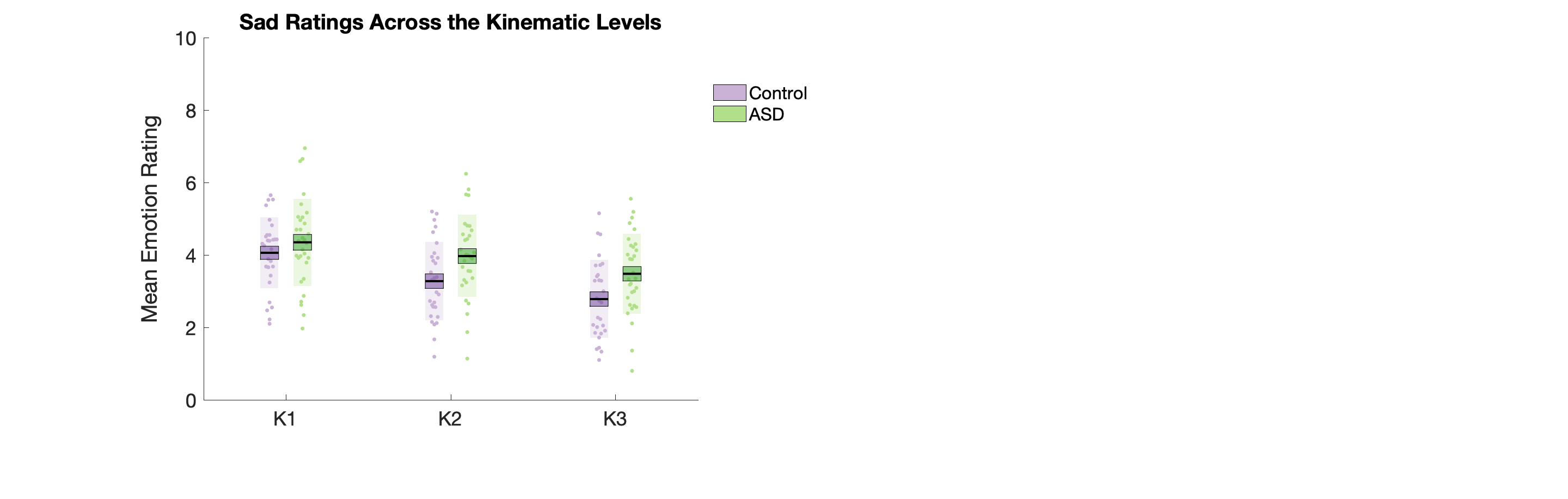


**Fig. 4** Mean sad ratings given by autistic and control participants across the kinematic levels (averaging across the displayed emotions and spatial levels). The black line represents the mean, the shaded region represents the standard deviation, the colored box represents 1 standard error around the mean and the dots are individual datapoints

In order to unpack the significant spatial x kinematic x rating x group interaction [F(8,464) = 2.76, p < .05, *η_P_^2^* = .05], we conducted post-hoc 2 x 3 x 3 ANOVAs (group x kinematic x rating) for each spatial level. This revealed a significant group x kinematic x rating interaction at the S2 [F(4,232) = 28.10, p < .001, *η_P_^2^* = .06] and S3 [F(4,232) = 2.46, p < .05, *η_P_^2^* = .04] level, but not at the S1 [p = .108] level. We then completed post-hoc 2 x 3 ANOVAs (group x kinematic) for each rating at the S2 and S3 level. At the S2 level we found a significant kinematic x group interaction for angry [F(2,116) = 4.55, p < .05, *η_P_^2^* = .07] and happy [F(2,116) = 3.97, p < .05, *η_P_^2^* = .06] ratings but not sad [p = .255] ratings. Whilst there was a significant effect of the kinematic manipulation on angry ratings at the S2 level for control participants (regardless of which emotion was displayed) [F(2,56) = 19.94, p < .001, *η_P_^2^* = .42], there was not a significant effect for autistic participants [p =.054] (see **Fig. 5**) (however, there were no group differences in angry ratings at the S2 level across each of the kinematic levels). In addition, we found that whilst there was a significant effect of the kinematic manipulation on happy ratings at the S2 level for autistic participants [F(2,60) = 8.88, p < .01, *η_P_^2^* = .23], there was not for controls [p= .424]. This difference in happy ratings across the kinematic levels led to a significant group difference, with autistic participants giving significantly higher happy ratings at the K3 [t(58) = -2.85, p_bonf_ < .05, mean difference = -0.80] but not K1 [p = .421] or K2 [p = .178] level (see **Fig. 6**).


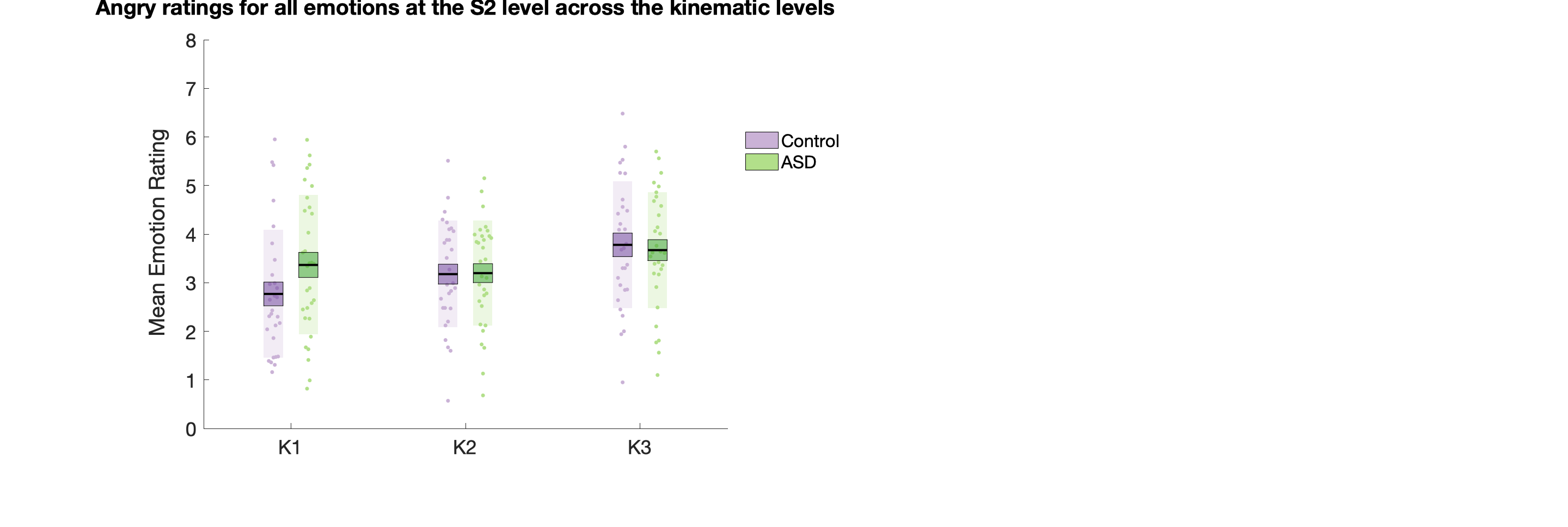


**Fig. 5** Mean angry ratings at the S2 level given by autistic and control participants across the kinematic levels (averaging across the displayed emotions). The black line represents the mean, the shaded region represents the standard deviation, the colored box represents 1 standard error around the mean and the dots are individual datapoints

**Fig. 6** Mean happy ratings at the S2 level given by autistic and control participants across the kinematic levels (averaging across the displayed emotions). The black line represents the mean, the shaded region represents the standard deviation, the colored box represents 1 standard error around the mean and the dots are individual datapoints

At the S3 level, we found a significant kinematic x group interaction for sad [F(2,116) = 3.80, p < .05**,** *η_P_^2^* = .06], but not angry [p = .129] or happy [p = .177] ratings. Whilst there was a significant effect of the kinematic manipulation on sad ratings at the S3 level for both control [F(2,56) = 34.02, p < .001, *η_P_^2^* = .55], and autistic [F(2,60) = 7.23, p < .01, *η_P_^2^* = .19] participants, the effect of the kinematic manipulation was greater for controls (see **Fig. 7.**). In other words, regardless of what emotion was displayed, at the S3 level, the sad ratings given by autistic (relative to control) participants were less affected by the kinematic manipulation. This smaller effect led to significant group differences in sad ratings, with autistic participants giving higher ratings (after Bonferroni-correction) at the K2 [t(58) = -22.57, p_bonf_ < .05, mean difference = -0.83] and K3 [t(58) = -3.35, p_bonf_ < .01, mean difference = -1.01] level, but not the K1 [p = .166] level. In our 2 x 3 ANOVA (group x kinematic) for sad ratings at the S3 level, we also identified a main effect of group, with autistic participants giving higher mean sad ratings at the S3 level (regardless of what emotion was displayed) [t(58) = -2.671, p < .05, mean difference = -0.75].

**Fig. 7** Mean sad ratings at the S3 level given by autistic and control participants across the kinematic levels (averaging across the displayed emotions). The black line represents the mean, the shaded region represents the standard deviation, the colored box represents 1 standard error around the mean and the dots are individual datapoints
